# Clinical and MRI Features of Posterior Reversible Encephalopathy Syndrome With Atypical Regions: A Descriptive Study With a Large Sample Size

**DOI:** 10.3389/fneur.2020.00194

**Published:** 2020-03-24

**Authors:** Kunhua Li, Yang Yang, Dajing Guo, Dong Sun, Chuanming Li

**Affiliations:** ^1^Department of Radiology, The Second Affiliated Hospital of Chongqing Medical University, Chongqing, China; ^2^Department of Radiology, Chongqing Prevention and Treatment Center for Occupational Diseases, Chongqing, China

**Keywords:** posterior reversible encephalopathy syndrome, clinical features, magnetic resonance imaging, atypical regions, vasogenic edema

## Abstract

**Background:** Accurate diagnosis and timely treatment for posterior reversible encephalopathy syndrome (PRES) with atypical regions are very important in clinical practice. However, until now, little has been known about the clinical and MRI manifestations of this disease. Therefore, the aim of this study is to investigate the clinical and MRI features of PRES to promote clinical management and deepen our understanding of this disease.

**Materials and Methods:** Data from six PRES patients with atypical regions were collected from our hospital. Data from another 550 cases were obtained by searching the PubMed, EMBASE and Web of Science databases with the keywords “posterior reversible encephalopathy syndrome” “PRES” “reversible posterior leukoencephalopathy” “RPLS” “hypertensive encephalopathy” “hyperperfusion encephalopathy” or “reversible posterior cerebral edema encephalopathy.” The clinical and MRI features of these 556 cases were analyzed together.

**Results:** A total of 305 patients were female, and 248 were male, with a median age of 34 years. The information on sex and age of three patients was not available. The most common symptom was headache (282/556, 50.7%), followed by altered mental status (243/556, 43.7%), seizures (233/556, 41.9%), visual disturbances (194/556, 34.9%), nausea/vomiting (130/556, 23.4%), and focal neurological deficits (101/556, 18.2%). Hypertension (425/556, 76.4%), renal diseases (152/556, 27.3%), immunosuppressant drugs (79/556, 14.2%), and chemotherapy/chemoradiotherapy (59/556, 10.6%) were the major predisposing factors. The atypical regions of the lesions were the cerebellum (331/556, 59.5%), basal ganglia (135/556, 24.3%), periventricular/deep white matter (125/556, 22.5%), pons (124/556, 22.3%), brainstem (115/556, 20.7%), thalamus (114/556, 20.5%), midbrain (48/556, 8.6%), spinal cord (33/556, 5.9%), and medulla (29/556, 5.2%). Additionally, the following typical regions were observed: occipital (278/556, 50.0%), parietal (234/556, 42.1%), frontal (150/556, 27.0%), and temporal (124/556, 22.3%) lobes. The major treatments were antihypertensives (358/515, 69.5%), antiepileptics/sedation (126/515, 24.5%), discontinuation/switching agents (67/515, 13.0%), and steroids (54/515, 10.5%). The median time of the clinical state improved and abnormal neuroimaging resolved is 2–3 weeks after appropriate treatment.

**Conclusion:** The common symptoms of PRES with atypical regions include headaches, altered mental status, seizures, visual disturbances, nausea or vomiting, and focal neurological deficits. The frequent predisposing factors include hypertension, renal diseases, immunosuppressant drugs and chemotherapy/chemoradiotherapy. MRI features are mainly characterized by vasogenic edema in central zones always accompanied by typical regions. Most cases can be reversed in 2–3 weeks when promptly recognized and properly treated.

## Introduction

Posterior reversible encephalopathy syndrome (PRES) is a reversible clinico-radiological entity associated with various conditions (e.g., renal failure, blood pressure fluctuations, cytotoxic drugs, autoimmune disorders, and pre-eclampsia or eclampsia), and the diverse clinical manifestations mainly include acute and subacute onset of headache, nausea, vomiting, seizures, altered mental status, visual disturbances, and focal neurological signs (1–4). The typical MRI feature of PRES is characterized by reversible vasogenic edema affecting the subcortical white matter of supratentorial lobes, especially in the parieto-occipital lobes (5). When promptly diagnosed and properly treated, the clinical and radiological abnormalities associated with PRES can be reversed entirely. Otherwise, some patients can progress to having hemorrhage, ischemia, massive infarction, and even death (6–8). Therefore, prompt identification of PRES is very important for the treatment and outcome of patients.

Previous studies have mostly focused on typical or classical PRES with three primary variations: a dominant parieto-occipital pattern, holohemispheric watershed pattern, and superior frontal sulcus pattern (5, 9). However, with the deepening of research on this disease in recent years, lesions have also been found to occur in atypical regions, such as the frontal lobe, thalamus, periventricular white matter, brainstem, cerebellum, and spinal cord, which are poorly understood and easily misdiagnosed (9–16). Therefore, it is very important to study the clinical and MRI features of PRES with atypical regions to improve clinical management. However, to our knowledge, most of the previous studies of PRES with atypical regions have been case reports or small case series lacking a comprehensive summary with a large sample (4, 17). Therefore, in this study, we investigate the clinical and MRI features of PRES with atypical regions in a large sample by retrospectively collecting data from patients in our hospital and from the patients reported in the literature by searching the PubMed, EMBASE, and Web of Science databases.

## Materials and Methods

This study was approved by the Ethics Committee of our institution. The requirement for informed consent was waived.

### Subjects

**Figure 1:**
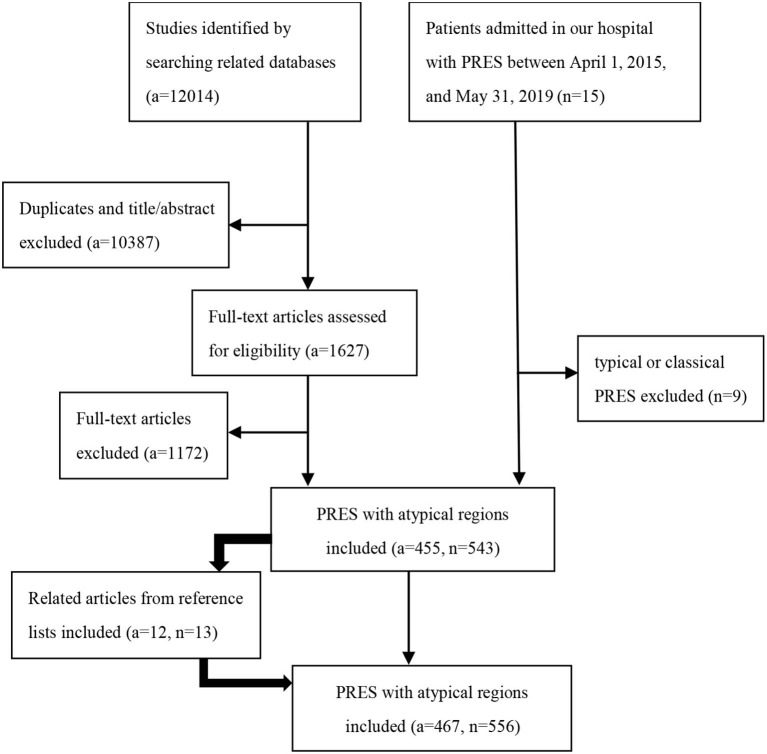
Flowchart of the study population. a, number of articles; n, number of patients.

We retrospectively collected patient information in two ways. (1) We searched the medical records of patients admitted to our hospital with PRES between April 1, 2015, and May 31, 2019. The diagnostic criteria used for PRES were previously described (5). (2) We searched the PubMed, EMBASE and Web of Science databases for articles published until May 31, 2019, with the keywords “posterior reversible encephalopathy syndrome” “PRES” “reversible posterior leukoencephalopathy” “RPLS” “hypertensive encephalopathy” “hyperperfusion encephalopathy” or “reversible posterior cerebral edema encephalopathy” (Table E1 in Supplementary Material). Additionally, we identified related articles through searches of the reference lists from the articles extracted from the searched files as supplements. Two authors (Kunhua Li and Yang Yang) independently reviewed the full-text articles of the relevant publications. In cases where there was ambiguity in opinions, a third author (Chuanming Li) made the final arbitration. The inclusion criteria of our study were as follows: (1) all patients underwent a minimum of fluid-attenuated inversion recovery (FLAIR), T2-weighted imaging (T2WI), and T1-weighted imaging (T1WI); (2) atypical involvements including the basal ganglia, thalamus, periventricular or deep white matter, cerebellum, brainstem, midbrain, pons, medulla oblongata, and spinal cord; and (3) only studies reported in English were included. The exclusion criteria were as follows: typical or classical PRES with three primary variations: a dominant parieto-occipital pattern, holohemispheric watershed pattern, and superior frontal sulcus pattern (9). The flowchart of the study population is shown in Figure 1.

### Clinical Evaluation

The clinical information collected and evaluated from the patient records included age, sex, predisposing factors for the development of PRES, presenting blood pressure, related symptoms, current drugs/therapies, follow-up interval and outcome.

### Imaging Evaluation

The imaging findings were evaluated on T1WI, T2WI, and FLAIR images in all cases. Diffusion-weighted imaging (DWI), apparent diffusion coefficient (ADC) maps, susceptibility-weighted imaging (SWI) or T2^*^-weighted gradient-echo imaging (T2^*^WI), gadolinium-enhanced T1WI, MR angiography (MRA), MR venography (MRV), and other advanced images were evaluated if they were available. DWI and ADC maps were analyzed to determine the vasogenic or cytotoxic edema in the lesions. SWI or T2^*^WI was used to determine the intracranial hemorrhage and microbleeds.

### Statistical Analysis

General demographic, clinical and MRI indicators were expressed as the mean ± SD (normally distributed quantitative variables), median (non-normally distributed quantitative variables), or numbers and percentages (categorical variables) for descriptive analysis.

## Results

**Figure 2:**
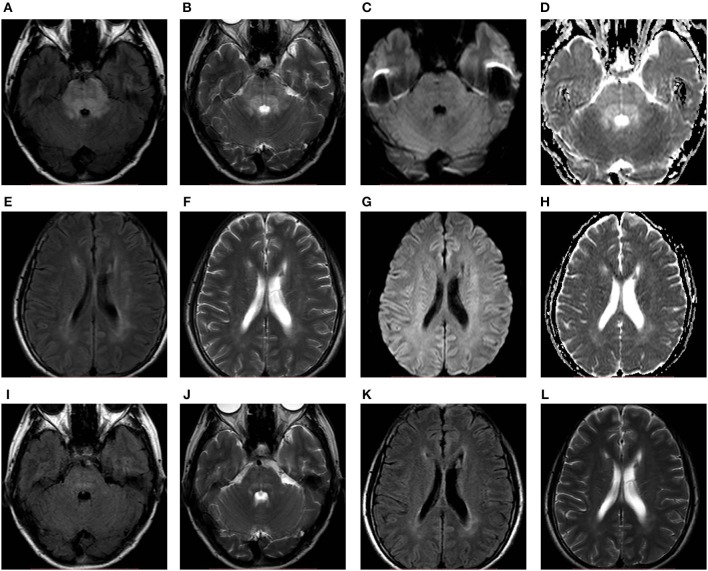
A 52-years-old female with hypertension presented with headache. FLAIR **(A,E)**, T2WI **(B,F)**, and ADC maps **(D,H)** showed hyperintensity predominantly in the brainstem accompanied by periventricular white matter. No obvious abnormality on DWI **(C,G)**. After 11 days of follow-up, the abnormal signals **(I–L)** markedly resolved. ADC, apparent diffusion coefficient; DWI, diffusion-weighted imaging; FLAIR, fluid-attenuated inversion recovery; T2WI, T2-weighted imaging.

In total, six patients from our hospital (the clinical and MRI features of six patients are shown in Table 1, and the MRI features of one patient are shown in Figure 2) and 550 patients from 467 articles published on PubMed, EMBASE and Web of Science met our inclusion criteria. All the articles ultimately included are shown in the List E1 in Supplementary Material.

**Table 1:** Demographic, clinical and MRI features of six patients in our hospital.

**Case No**.	**Age (years)/sex**	**Symptoms**	**Blood pressure (mmHg)**	**Predisposing factors**	**Location**	**Hemorrhage**	**Acute infarction**	**Treatment**
1	52/F	Headache	200/130	Hypertension	Brainstem, periventricular	–	–	Antihypertensive
2	23/F	Focal neurological deficits	163/116	Preeclampsia	Brainstem, periventricular	–	Pons (+)	Antihypertensive
3	40/M	Focal neurological deficits	189/110	Hypertension, psoriasis	Periventricular, basal ganglia, pons, cerebellum	Pons (+)	–	Antihypertensive
4	44/F	Focal neurological deficits	260/130	Hypertension, renal dysfunction, renal artery stenosis	Brainstem, periventricular	–	Cerebrum (+)	Antihypertensive
5	45/F	Insomnia	236/154	Hypertension, renal dysfunction	Periventricular, basal ganglia, pons	–	–	Antihypertensive
6	22/F	Headache, blurred vision	224/115	Hypertension	Periventricular, basal ganglia	–	–	Antihypertensive

### Clinical Features

A total of 305 patients were females, and 248 were males, with a median age of 34 years. The information regarding sex and age of three patients was not available in the descriptions in the literature. The most common symptom was headache (282/556, 50.7%), followed by altered mental status (243/556, 43.7%), seizure (233/556, 41.9%), visual disturbance (194/556, 34.9%), nausea/vomiting (130/556, 23.4%), and focal neurological deficit (104/556, 18.7%) in descending order. Other rare symptoms are shown in Table 2. Hypertension (425/556, 76.4%), renal diseases (152/556, 27.3%), immunosuppressant drugs (79/556, 14.2%), and chemotherapy/chemoradiotherapy (59/556, 10.6%) were the major predisposing factors. For patients with hypertension, the median systolic blood pressure was 200 mm Hg (range, 120–292 mm Hg), and the median diastolic blood pressure was 118 mm Hg (67–220 mm Hg). The median arterial pressure (MAP) was 143 mmHg (100–237 mmHg). The demographics and clinical characteristics of PRES patients with atypical regions are summarized in Table 2.

**Table 2:** Demographic and clinical characteristic of PRES patients with atypical regions.

**Characteristic**	***n***
**DEMOGRAPHICS AND SYMPTOMS**	
Sex/F	305/553 (55.2%)
Age (median, range, years)	(34, 0.08–85)
**Headache**	**282/556 (50.7%)**
**Altered mental status**	**243/556 (43.7%)**
**Seizures**	**233/556 (41.9%)**
**Visual disturbances**	**194/556 (34.9%)**
**Nausea/vomiting**	**130/556 (23.4%)**
**Focal neurological deficits**	**101/556 (18.2%)**
Dizziness	38/556 (6.8%)
Gait disturbances	27/556 (4.9%)
Fever	25/556 (4.5%)
Disorientation	23/556 (4.1%)
Ataxia	20/556 (3.6%)
Dyspnea	16/556 (2.9%)
Abdominal pain	13/556 (2.3%)
Abnormal urine	12/556 (2.2%)
Others (each symptom)^#^	≤2%
**PREDISPOSING FACTOR**
**Hypertension**	**425/556 (76.4%)**
**Renal diseases**	**152/556 (27.3%)**
**Immunosuppressant drugs**	**79/556 (14.2%)**
**Chemotherapy/chemoradiotherapy**	**59/556 (10.6%)**
Autoimmune disorders	55/556 (9.9%)
Pre-eclampsia/Eclampsia	41/556 (7.4%)
Infection/sepsis/shock	32/556 (5.8%)
Steroids	24/556 (4.3%)
Metabolic disorders	15/556 (2.7%)
Miscellaneous drugs	13/556 (2.3%)
Dialysis	12/556 (2.2%)
Transfusion	11/556 (2.0%)
Endocrine disorders	7/556 (1.3%)
Surgery	6/556 (1.1%)
Others (each factor)^*^	≤1%
**TREATMENT**	
**Antihypertensives**	**358/515 (69.5%)**
**Antiepileptics/sedation**	**126/515 (24.5%)**
**Discontinuation/switching agents**	**67/515 (13.0%)**
**Steroid**	**54/515 (10.5%)**
Dehydrating/diuretics	34/515 (6.6%)
Intracranial decompression	24/515 (4.7%)
Hemodialysis	23/515 (4.5%)
Immunosuppressive therapy	20/515 (3.9%)
Anti-infective treatment	16/515 (3.1%)
Others (each treatment)^$^	≤2%

*The main characteristics (>10%) are marked in bold*.

*Others (each symptom)^#^: involuntary movement, fatigue, behavioral changes, edema, loss of appetite, neck stiffness, diarrhea, polydipsia and weight loss, purpura, and insomnia*.

Others (each factor)^*^: sickle cell disease, substance abuser, reduction in intracranial pressure, intoxication, contrast medium exposure, trauma, multiple system atrophy, embolus, hyperbaric oxygen therapy.

*Others (each treatment)^$^: plasma exchange, treatment for tumor, renal angioplasty, glyceryl trinitrate infusion, and others <1%*.

### Imaging Features

All patients showed hyperintensity signals on T2WI and FLAIR images. The atypical regions of the lesions were the cerebellum (331/556, 59.5%), basal ganglia (135/556, 24.3%), periventricular/deep white matter (125/556, 22.5%), pons (124/556, 22.3%), brainstem (115/556, 20.7%), thalamus (114/556, 20.5%), midbrain (48/556, 8.6%), spinal cord (33/556, 5.9%) and medulla (29/556, 5.2%). Additionally, the following typical regions were observed: occipital (278/556, 50.0%), parietal (234/556, 42.1%), frontal (150/556, 27.0%), and temporal (124/556, 22.3%). A total of 148 patients had DWI and ADC maps, and 34 (23.0%) patients showed cytotoxic edema on the background of vasogenic edema. Thirty-three (5.9%) and 35 (6.3%) patients had intracranial hemorrhage and hydrocephalus, respectively. Thirty-one patients had acute infarcts. Ninety-three patients underwent gadolinium-enhanced T1WI, and 29 (31.2%) patients showed lesion enhancements. Twenty-four (4.3%) patients in our study underwent SWI or T2^*^WI examination, and 79.2% (19/24) of patients were confirmed to have microbleeds based on SWI or T2^*^WI. The MRI characteristics of PRES with atypical regions are summarized in Table 3. Sixty-two patients in our study underwent MRA examination, which suggested stenosis/occlusion (8/62, 12.9%), vasospasm (6/62, 9.7%), aneurysm (3/62, 4.8%), hypoplasia (2/62, 3.2%), and dilatation (1/62, 1.6%). Fifteen patients in our study underwent MRV examination, which suggested hypoplasia (2/15, 13.3%) and thrombosis/stenosis (1/15, 6.7%). The main MRI characteristics of PRES patients with atypical regions are summarized in Table 3.

**Table 3:** MR characteristics of PRES patients with atypical regions.

**Location**		**MR feature**	
Cerebellum	331/556 (59.5%)	T1WI(–)&T2WI(+)	556/556 (100.0%)
Occipital lobe	278/556 (50.0%)	DWI(=)&ADC(+)	47/148 (31.8%)
Parietal lobe	234/556 (42.1%)	DWI(+)&ADC(+)	43/148 (29.1%)
Frontal lobe	150/556 (27.0%)	DWI(+)&ADC(–)	31/148 (20.9%)
Basal ganglia	135/556 (24.3%)	DWI(=)&ADC(=)	20/148 (13.5%)
Periventricular/deep white matter	125/556 (22.5%)	DWI(+)&ADC(=)	5/148 (3.4%)
Temporal lobe	124/556 (22.3%)	DWI(–)&ADC(–)	3/148 (2.0%)
Pons	124/556 (22.3%)	DWI(–)&ADC(+)	2/148 (1.4%)
Brainstem	115/556 (20.7%)	DWI(–)&ADC(=)	1/148 (0.7%)
Thalamus	114/556 (20.5%)	Enhancement	29/93 (31.2%)
Midbrain	48/556 (8.6%)	Hemorrhage	33/556 (5.9%)
Spinal cord	33/556 (5.9%)	Microbleeds	19/24 (79.2%)
Medulla	29/556 (5.2%)	Hydrocephalus	35/556 (6.3%)

*ADC, apparent diffusion coefficient; DWI, diffusion-weighted imaging; T1WI, T1-weighted imaging; T2WI, T2-weighted imaging; –, hypointensity; =, iso-intensity; +, hyperintensity*.

### Treatment and Outcome

The details of the treatment were available in 515 cases. The major treatments were antihypertensives (358/515, 69.5%), antiepileptics/sedation (126/515, 24.5%), discontinuation/switching agents (67/515, 13.0%), and steroids (54/515, 10.5%). After appropriate treatments, the neurological symptoms of 244 patients resolved at follow-up [median time, 14 days (range, 0.04–540 days)]. Twenty-five patients died at follow-up; however, most of their deaths (20/25, 80.0%) were not attributable to PRES but to severe infections or malignant tumors. Moreover, the causes of the three patients' deaths were unknown. In 364 patients, data on follow-up time and MR imaging were available. Except for four patients with no significant change, the MRIs of 360 patients at follow-up showed lesion reversal [complete, 273 patients, median time, 21 days (range, 1–720 days); partial, 87 patients, median time, 18 days (0.5–300 days)].

## Discussion

PRES with atypical regions can be easily misdiagnosed, which can lead to a delay or wrong choice of management and subsequent irreversible injury. Thus, it is crucial for clinicians to improve their understanding of the clinical and MRI features of this disease (12, 18). To our knowledge, this is the first comprehensive study with a large sample of PRES patients with atypical regions.

Recognition of the clinical features of this disease is important for prompt diagnosis and rational management. In our study, most patients were young females, which is similar to most previous studies, but males were predominant in some other studies (12, 13). This may be due to different sample sizes and inclusion criteria. We found that the common clinical symptoms included headache, altered mental status, seizures, visual disturbances, nausea/vomiting and focal neurological deficits. Many patients showed several different symptoms concurrently or subsequently. However, the symptoms often did not correspond to the brain lesion locations. For example, 20.7% (115/556) of patients had brainstem lesions, but most of them did not have specific brainstem signs. This may suggest that there is no obvious association between brain lesions and clinical manifestations in this disease (11, 18, 19). The frequent predisposing factors were those classically described, namely, hypertension, renal diseases, immunosuppressant drugs, and chemotherapy/chemoradiotherapy. Acute or severe hypertension occurred in 76.4% of patients in our study, which may be explained by cerebral autoregulation impairment as the primary pathogenic mechanism in PRES (4, 5, 20, 21). Previous studies have reported that the proportion of hypertension in patients with PRES ranges from 20 to 65% (4). Our results of a proportion of 76.4% are slightly higher, possibly because the central variant of PRES may be a higher incidence of hypertension (22), which had a higher incidence in our study. Nevertheless, 23.6% of patients still developed PRES without hypertension. All of them had other predisposing factors of immunosuppressant drugs, autoimmune disorders, chemotherapy or infection/sepsis/shock. These predisposing factors may induce endothelial damage or dysfunction, resulting in vasogenic edema and PRES (23–27).

In addition to the clinical features, neuroimaging, especially MRI, is essential in the evaluation and diagnosis of PRES with atypical regions (28). The lesion locations are very important in terms of the MRI features. Atypical region involvement mostly occurs in central zones (such as the basal ganglia, thalami, periventricular or deep white matter, brainstem and spinal cord). Compared with previous studies (9, 11, 16, 29), in our study, there were some similar locations but with different incidences; this may be due to the different sample sizes and populations. We focused on PRES with atypical region involvement and used a large sample. Although we excluded the three primary variations of typical PRES, we found that the occipital (278, 50.0%), parietal (234, 42.1%), and frontal (150, 27.0%) lobes were still commonly involved. This suggests that PRES with atypical region involvement is often accompanied by typical region involvement (3, 29).

Vasogenic edema, which is an essential pathological feature of PRES, is usually hypointense on T1WI, hyperintense on T2WI and FLAIR, and isointense or hyperintense on DWI and ADC maps. Hyperintensity on DWI and hypointensity on ADC maps, which are called restricted diffusion, can reflect cytotoxic edema. The presence of cytotoxic edema may suggest progression to infarction and eventual irreversible damage, which may be associated with poor outcome (30, 31). In our study, 34 (23.0%) patients showed cytotoxic edema, but only two patients had a residual infarction. This may be because most of the patients only had small areas of cytotoxic edema within the predominant backgrounds of vasogenic edema. Contrast enhancement is not necessary for the diagnosis of PRES but may be useful for the exclusion of other clinical considerations (8, 32). In this study, we found that 31.2% of patients showed lesion enhancement. The enhancement may have been induced by the breakdown of the blood-brain barrier, which is related to endothelial injury or dysfunction (33, 34). The rates of enhancement vary within the previous literature, ranging from 23.1 to 43.7% in PRES (29, 34, 35), likely related to differences in timing, magnetic field strength, and contrast agent dose/relaxivity.

In addition to the common MRI features, concomitant and coincidental events that occur on neuroimaging, mainly including hemorrhage, microbleeds and hydrocephalus, can occur in PRES. In our study, hemorrhage was found in 33 (5.9%) patients. The incidence rate was lower than that of previous SWI or T2^*^WI studies, where it ranges from 15 to 65% (30, 36–38). The possible reason is that SWI or T2^*^WI is more sensitive to hemorrhage than conventional MRI, and the previous literature has shown a higher incidence of hemorrhage with SWI or T2^*^WI examination. The fact that only 24 (4.3%) patients in our study with SWI or T2^*^WI examination supports this hypothesis. Thirty-five (6.3%) patients had obstructive hydrocephalus due to infratentorial involvement, especially of the cerebellum, which was caused by the compression of adjacent swollen brain tissue.

Once PRES has been diagnosed, the treatment, which mainly includes supportive treatment and the elimination of the cause, should be undertaken immediately to prevent poor progression. In our study, 69.5% of patients received antihypertensive treatment, and 24.5% of patients received antiepileptics/sedation. After proper and prompt treatments, the clinical state improved, and abnormal neuroimaging resolved in most patients within 2–3 weeks.

## Conclusions

In conclusion, we found that PRES with atypical regions had diverse clinical and MRI features. The common symptoms of this disease include headache, altered mental status, seizure, visual disturbances, nausea or vomiting, and focal neurological deficits; the frequent predisposing factors include hypertension, renal diseases, immunosuppressant drugs, and chemotherapy/chemoradiotherapy; and the MRI features are mainly characterized by vasogenic edema in central zones (such as the basal ganglia, thalami, periventricular or deep white matter, brainstem, and spinal cord) always accompanied by abnormalities in typical regions. Most lesions are reversed in 2–3 weeks when promptly recognized and properly treated. The main limitation of this study is the possible selection bias because only publications in English were searched and included. This aspect needs to be improved in future research.

## Data Availability Statement

All datasets generated for this study are included in the article/Supplementary Material.

## Ethics Statement

The studies involving human participants were reviewed and approved by the Ethics Committee of The Second Affiliated Hospital of Chongqing Medical University. Written informed consent from the participants' legal guardian/next of kin was not required to participate in this study in accordance with the national legislation and the institutional requirements.

## Author Contributions

KL, YY, DG, and CL contributed the conception and design of the study. KL and YY organized the database and performed the statistical analysis. KL, YY, and DS wrote the first draft of the manuscript. All authors contributed to the manuscript revision, read, and approved the submitted version.

### Conflict of Interest

The authors declare that the research was conducted in the absence of any commercial or financial relationships that could be construed as a potential conflict of interest.
