# Supplementary material for: Clinical and MRI Features of Posterior Reversible Encephalopathy Syndrome With Atypical Regions: A Descriptive Study With a Large Sample Size
Source: Front Neurol. 2020 Mar 24;11:194. doi: 10.3389/fneur.2020.00194 (PMC7105821; doi:10.3389/fneur.2020.00194)
Supplement: Supplementary file 1 [file Table_1.docx]

**Table E1:** Used Keywords for Literature Search

| Databases | PubMed, EMBASE and Web of Science |
| --- | --- |
| Search terms | “posterior reversible encephalopathy syndrome” “PRES” “reversible posterior leukoencephalopathy” “RPLS” “hypertensive encephalopathy” “hyperperfusion encephalopathy” or “reversible posterior cerebral edema encephalopathy” |

**List E1:** 467 articles finally included were shown as follow:(1-155)(156-331)(332-467)

1. Yang Q, Chang CC, Liu M, Yu YQ. Sequential occurrence of eclampsia-associated posterior reversible encephalopathy syndrome and reversible splenial lesion syndrome (a case report): proposal of a novel pathogenesis for reversible splenial lesion syndrome. BMC medical imaging 2019;19(1):35. doi: 10.1186/s12880-019-0323-7

2. Wijenayake Galagamage IDK, Sujith A, Kiringodage AK. Isolated pontine involvement in posterior reversible encephalopathy syndrome with coincidental acute ischaemic stroke. BMJ case reports 2019;12(4). doi: 10.1136/bcr-2018-227132

3. Vitucci A, Lojacono A, Gatti E, Prefumo F, Fratelli N. Atypical presentation and imaging features of postpartum posterior reversible encephalopathy syndrome. Journal of obstetrics and gynaecology : the journal of the Institute of Obstetrics and Gynaecology 2019;39(3):412-414. doi: 10.1080/01443615.2018.1474188

4. Vaysman T, Xu P, Vartanian T, Michalak P, Pike K, Liu A. "Highlighting" red nuclei by atypical posterior reversible encephalopathy syndrome in a patient with systemic lupus erythematosus. Clinical case reports 2019;7(7):1404-1408. doi: 10.1002/ccr3.2245

5. Shen T, Chen H, Jing J, Raza HK, Zhang Z, Bao L, Zhou S, Zhang S, Cui G. A study on clinical characteristics and the causes of missed diagnosis of reversible posterior leukoencephalopathy syndrome in eclampsia. Neurological sciences : official journal of the Italian Neurological Society and of the Italian Society of Clinical Neurophysiology 2019. doi: 10.1007/s10072-019-03914-3

6. Sarbu MI, Sarbu N. Central PRES (posterior reversible encephalopathy syndrome) in HELLP syndrome. Internal and Emergency Medicine 2019;14(4):617-618. doi: 10.1007/s11739-018-2003-y

7. Samara A, Berry B, Ghannam M. Posterior reversible encephalopathy syndrome with isolated infratentorial involvement: A case report. Radiology case reports 2019;14(5):576-580. doi: 10.1016/j.radcr.2019.02.009

8. Saleemi MA, Desai T. Posterior reversible encephalopathy syndrome affecting the entire neuraxis. Journal of Neuroimaging 2019;29(2):276-277.

9. Marcoccia E, Piccioni MG, Schiavi MC, Colagiovanni V, Zannini I, Musella A, Visentin VS, Vena F, Masselli G, Monti M, Perrone G, Panici PB, Brunelli R. Postpartum Posterior Reversible Encephalopathy Syndrome (PRES): Three Case Reports and Literature Review. Case reports in obstetrics and gynecology 2019;2019:9527632. doi: 10.1155/2019/9527632

10. Liu L, Dai D, Cao F, Zhang L, Wang X. Posterior reversible encephalopathy syndrome with spinal cord involvement but without hemisphere lesions: A case report. Medicine 2019;98(2):e13649. doi: 10.1097/md.0000000000013649

11. Karadan U, Naga SR, Chellenton J, Ummer A, Muthyala PR. Bilateral lateral geniculate body lesions causing reversible blindness in a patient with posterior reversible encephalopathy syndrome. The journal of the Royal College of Physicians of Edinburgh 2019;49(1):34-36. doi: 10.4997/jrcpe.2019.107

12. Kaneko S, Hirai K, Minato S, Yanai K, Mutsuyoshi Y, Ishii H, Kitano T, Shindo M, Aomatsu A, Miyazawa H, Ito K, Ueda Y, Hoshino T, Ookawara S, Morishita Y. A case of posterior reversible encephalopathy syndrome in a patient undergoing automated peritoneal dialysis. CEN case reports 2019. doi: 10.1007/s13730-019-00389-1

13. Kamo H, Ueno Y, Sugiyama M, Miyamoto N, Yamashiro K, Tanaka R, Yokoyama K, Hattori N. Pontine hemorrhage accompanied by neuromyelitis optica spectrum disorder. Journal of neuroimmunology 2019;330:19-22. doi: 10.1016/j.jneuroim.2019.01.020

14. Hubbard ME, Phillips AA, Charbonneau R, Squair JW, Parr AM, Krassioukov A. PRES secondary to autonomic dysreflexia: A case series and review of the literature. The journal of spinal cord medicine 2019:1-7. doi: 10.1080/10790268.2019.1616146

15. Hebant B, Guegan-Massardier E, Triquenot-Bagan A, Ozkul-Wermester O. Atypical MRI presentation of posterior reversible encephalopathy syndrome with predominant brainstem involvement. Acta neurologica Belgica 2019;119(1):123-125. doi: 10.1007/s13760-018-1055-0

16. Di Giuliano F, Picchi E, Scaggiante J, Ferrante P, Misciasci T, Da Ros V, Pistolese CA, Floris R, Garaci F. Posterior reversible encephalopathy syndrome and Wernicke encephalopathy in patient with acute graft-versus-host disease. Radiology case reports 2019;14(8):971-976. doi: 10.1016/j.radcr.2019.05.024

17. Dhawan SR, Goswami JN, Suthar R, Dayal D, Vyas S, Singhi PD. A Child with Central Variant Posterior Reversible Encephalopathy Syndrome. Neuropediatrics 2019;50(1):66-67. doi: 10.1055/s-0038-1675237

18. Dalli T, Dalli S, Galea R, Chircop C. Posterior reversible encephalopathy syndrome. European Journal of Obstetrics & Gynecology and Reproductive Biology 2019;234:e188. doi: 10.1016/j.ejogrb.2018.08.040

19. Benetou CB, Siddiqui AS, Reid CR, Lim MJL. Cerebro-spinal posterior reversible encephalopathy syndrome (PRES) mimicking acute disseminated encephalomyelitis (ADEM) in a neurologically asymptomatic 18-month old girl. Developmental Medicine and Child Neurology 2019;61:77. doi: 10.1111/dmcn.14120

20. Wittgrove C, Kaur H, Siddiqui JH. Atypical Variant of Posterior Reversible Encephalopathy Syndrome in the Setting of Renovascular Hypertension: Case Report and Review of Literature. Cureus 2018;10(11):e3573. doi: 10.7759/cureus.3573

21. Tiwana H, Elangovan C, Niazi M, Kumar A. Atypical Presentation of Posterior Reversible Encephalopathy Syndrome with Spinal Cord Lesion. Annals of Neurology 2018;84:S44-S44.

22. Sarbu MI, Sarbu N. Central PRES (posterior reversible encephalopathy syndrome) in HELLP syndrome. Intern Emerg Med 2018. doi: 10.1007/s11739-018-2003-y

23. Ross Russell AL, Prevett M, Cook P, Barker CS, Pinto AA. Reversible cerebellar oedema secondary to profound hypomagnesaemia. Practical neurology 2018;18(4):311-314. doi: 10.1136/practneurol-2017-001832

24. Ramgopal A, Thavamani A, Ghori A. Association between Posterior Reversible Encephalopathy Syndrome and Mycoplasma pneumoniae infection. Journal of pediatric neurosciences 2018;13(1):109-111. doi: 10.4103/jpn.Jpn_145_17

25. Kumar N, Singh R, Sharma N, Jain A. Atypical presentation of posterior reversible encephalopathy syndrome: Two cases. Journal of anaesthesiology, clinical pharmacology 2018;34(1):120-122. doi: 10.4103/0970-9185.173351

26. Ou S, Xia L, Wang L, Xia L, Zhou Q, Pan S. Posterior Reversible Encephalopathy Syndrome With Isolated Involving Infratentorial Structures. Frontiers in neurology 2018;9:843. doi: 10.3389/fneur.2018.00843

27. Omoto S, Utsumi T, Matsuno H, Terasawa Y, Iguchi Y. Thrombotic Microangiopathy Presenting with Intestinal Involvement Following Long-term Interferon-beta1b Treatment for Multiple Sclerosis. Internal medicine (Tokyo, Japan) 2018;57(5):741-744. doi: 10.2169/internalmedicine.9326-17

28. Ogura S, Narumiya H, Iiduka R, Nagakane Y. Late recovery from unconsciousness in a patient with severe posterior reversible encephalopathy syndrome. Clin Case Rep 2018;6(9):1825-1828. doi: 10.1002/ccr3.1740

29. Nguyen H. Posterior Reversible Encephalopathy Syndrome Associated with Pazopanib. Annals of Neurology 2018;84:S158-S158.

30. Nao J, Zhang H, Wu S, Zhang X, Zheng D. Posterior reversible encephalopathy syndrome with spinal cord involvement (PRES-SCI) as a rare complication of severe diabetic ketoacidosis: a case report and review of the literature. Child's nervous system : ChNS : official journal of the International Society for Pediatric Neurosurgery 2018;34(4):701-705. doi: 10.1007/s00381-018-3724-y

31. Nakamura Y, Sugino M, Tsukahara A, Nakazawa H, Yamamoto N, Arawaka S. Posterior reversible encephalopathy syndrome with extensive cytotoxic edema after blood transfusion: a case report and literature review. BMC neurology 2018;18(1):190. doi: 10.1186/s12883-018-1194-1

32. Moghaddam AS, Ramaiah G, Jumaa M, Zaidi S. Posterior Reversible Encephalopathy Syndrome (PRES) Secondary to Use of Axitinib in Renal Cell Cancer. Neurology 2018;90(15).

33. Malkan UY, Gunes G, Demiroglu H, Goker H. Immunosuppression-associated posterior reversible encephalopathy syndrome in an acute leukemia case. Hematology reports 2018;10(4):7257. doi: 10.4081/hr.2018.7257

34. Maier S, Monica C, Romaniuc A, Andone S, Balasa R. Central-variant posterior reversible encephalopathy syndrome in a young patient with systemic lupus erythematosus. Acta neurologica Belgica 2018. doi: 10.1007/s13760-018-1046-1

35. Mai NTH, Phu NH, Nghia HDT, Phuong TM, Duc DT, Chau NVV, Wills B, Lim CCT, Thwaites G, Simmons CP, Yacoub S. Dengue-Associated Posterior Reversible Encephalopathy Syndrome, Vietnam. Emerging infectious diseases 2018;24(2):402-404. doi: 10.3201/eid2402.171634

36. Liu C, Cao J, Su Z, Xu S. Isolated brainstem involvement in posterior reversible encephalopathy syndrome: a case report and review of the literature. The International journal of neuroscience 2018:1-6. doi: 10.1080/00207454.2018.1561452

37. Lin YJ, Huang YT, Po HL. Hypertensive encephalopathy and multiple ischemic stroke as initial presentation of fabry disease. European Stroke Journal 2018;3(1):427. doi: 10.1177/2396987318770127

38. Lin SF, Wu TI, Fang CL, Lee JE. Intraabdominal and Pelvic actinomycosis in association with posterior reversible encephalopathy syndrome (PRES): A case report. Journal of neuroradiology Journal de neuroradiologie 2018;45(4):262-264. doi: 10.1016/j.neurad.2018.04.007

39. Lee SW, Lee SJ. Central-Variant Posterior Reversible Encephalopathy Syndrome with Albuminocytologic Dissociation. Case reports in neurology 2018;10(1):29-33. doi: 10.1159/000486444

40. Koh YH. Heat Stroke with Status Epilepticus Secondary to Posterior Reversible Encephalopathy Syndrome (PRES). Case reports in critical care 2018;2018:3597474. doi: 10.1155/2018/3597474

41. Kim HG, Lee KM, Lee JS. Unusual magnetic resonance imaging findings in a patient with posterior reversible encephalopathy syndrome. Quantitative imaging in medicine and surgery 2018;8(10):1066-1068. doi: 10.21037/qims.2018.10.10

42. Kazahari S, Honma K, Kawamura R, Uesugi T, Nagata E, Takizawa S. Symptomatic Lacunar Infarct Accompanied with Posterior Reversible Encephalopathy Syndrome: A Case Report. The Tokai journal of experimental and clinical medicine 2018;43(2):64-67.

43. Iyer RS, Ramalingam RTC, Akhtar S, Muthukalathi K. Perimesencephalic and sulcal subarachnoid haemorrhage: an interesting presentation of posterior reversible encephalopathy syndrome. BMJ case reports 2018;2018. doi: 10.1136/bcr-2017-222875

44. Iftikhar H, Magsi S, Poddar V. Pres-Ed out of Vision: Case of Transient Vision Loss in Hypertensive Emergency. J Gen Intern Med 2018;33(2):S592-S593.

45. Gungor S, Kilic B, Tabel Y, Selimoglu A, Ozgen U, Yilmaz S. Clinical and Imaging Findings in Childhood Posterior Reversible Encephalopathy Syndrome. Iran J Child Neurol 2018;12(1):16-25. doi: 10.22037/ijcn.v12i1.12336

46. Garcha M, Sivakumar K, El-Hunjul M, Varade S, Yacoub HA. Intracranial hemorrhage in the setting of posterior reversible encephalopathy syndrome: two case reports and a review. Hospital practice (1995) 2018;46(3):103-109. doi: 10.1080/21548331.2018.1451204

47. Ganesh K, Nair RR, Kurian G, Mathew A, Sreedharan S, Paul Z. Posterior Reversible Encephalopathy Syndrome in Kidney Disease. Kidney international reports 2018;3(2):502-507. doi: 10.1016/j.ekir.2017.10.017

48. Deguchi S, Mitsuya K, Nakasu Y, Hayashi N, Katagiri H, Murata H, Wasa J, Takahashi M, Endo M. Posterior reversible encephalopathy syndrome (PRES) induced by pazopanib, a multi-targeting tyrosine kinase inhibitor, in a patient with soft-tissue sarcoma: case report and review of the literature. Investigational new drugs 2018;36(2):346-349. doi: 10.1007/s10637-017-0521-5

49. Chou MC, Lee CY, Chao SC. Temporary Visual Loss Due to Posterior Reversible Encephalopathy Syndrome in the Case of an End-Stage Renal Disease Patient. Neuro-ophthalmology (Aeolus Press) 2018;42(1):35-39. doi: 10.1080/01658107.2017.1322109

50. Chan PKJ, Tse KS, Fok WSE, Poon WL. First case of neurofibromatosis with posterior reversible encephalopathy syndrome showing spinal cord involvement. Indian Journal of Radiology and Imaging 2018;28(2):161-164. doi: 10.4103/ijri.IJRI_320_17

51. Cerrone P, Sucapane P, Totaro R, Sacco S, Carolei A, Marini C. Posterior Reversible Encephalopathy Syndrome Presenting with Atypical Findings: Report of Two Cases. Case reports in neurological medicine 2018;2018:7835415. doi: 10.1155/2018/7835415

52. Birner B, Hirzel C, Wagner F, Waldegg G. Posterior reversible encephalopathy syndrome in an HIV-infected patient on antiretroviral treatment: what is the risk factor? BMJ case reports 2018;2018. doi: 10.1136/bcr-2017-221998

53. Benatti C, Pavesi C, Di Donato C. Posterior reversible encephalopathy syndrome: A case report. Italian Journal of Medicine 2018;12(2):28. doi: 10.4081/itjm.2018.s2

54. Bandeo L, Rausch A, Saucedo M, Chertcoff A, Cejas LL, Roca CU, Pacha S, Pardal MF, Reisin R, Bonardo P. Convexity Subarachnoid Hemorrhage Secondary to Adalidumab in a Patient with Ulcerative Colitis. Journal of vascular and interventional neurology 2018;10(2):62-64.

55. Aureli V, Giammattei L, Maduri R, Daniel RT, Messerer M. Posterior reversible encephalopathy syndrome (PRES) due to neuroblastoma in a child presenting with acute hydrocephalus. Child's nervous system : ChNS : official journal of the International Society for Pediatric Neurosurgery 2018;34(1):15-17. doi: 10.1007/s00381-017-3640-6

56. Arslan H, Yavuz A, Arslan A, Aycan A. Posterior reversible encephalopathy syndrome in IgA vasculitis: Neuroimaging of a 14-year-old child. Neurologia i neurochirurgia polska 2018;52(1):107-111. doi: 10.1016/j.pjnns.2017.11.006

57. Almoussa M, Goertzen A, Brauckmann S, Fauser B, Zimmermann CW. Posterior Reversible Encephalopathy Syndrome due to Hypomagnesemia: A Case Report and Literature Review. Case reports in medicine 2018;2018:1980638. doi: 10.1155/2018/1980638

58. Alexandrou ME, Kyriklidou P, Manou E, Tsagkourias M, Rudolf J, Pateinakis P, Mplatsa A, Lysitska A, Mitsopoulos E, Matamis D, Papadopoulou D. Posterior reversible encephalopathy syndrome (PRES) following intravenous administration of cyclophosphamide in a granulomatosis with polyangitis patient. Nephrology Dialysis Transplantation 2018;33:i392. doi: 10.1093/ndt/gfy104.SP144

59. Abughanimeh OK, Qasrawi AH, Tahboub MY, Abu Ghanimeh MK. Posterior reversible encephalopathy syndrome while receiving irinotecan with fluorouracil and folinic acid for metastatic gastric cancer. BJR case reports 2018;4(1):20170033. doi: 10.1259/bjrcr.20170033

60. Xia L, Ou SC, Wang L, Pan SQ. Posterior reversible encephalopathy syndrome predominantly involving brainstem and spinal cord. Int J Clin Exp Med 2017;10(6):9708-9710.

61. Villelli NW, Prevedello DM, Ikeda DS, Montaser AS, Otto BA, Carrau RL. Posterior Reversible Encephalopathy Syndrome Causing Vision Loss After Endoscopic Endonasal Resection of Pituitary Adenoma. World neurosurgery 2017;100:708.e701-708.e710. doi: 10.1016/j.wneu.2017.02.050

62. Tetsuka S, Nonaka H. Importance of correctly interpreting magnetic resonance imaging to diagnose posterior reversible encephalopathy syndrome associated with HELLP syndrome: a case report. BMC medical imaging 2017;17(1):35. doi: 10.1186/s12880-017-0208-6

63. Srinivasan KG, Balasubramanian P, Mayilvaganan KR, Kannan UN, Bilal M. Central Variant of Posterior Reversible Encephalopathy Syndrome - A Rare Case Report. Journal of clinical and diagnostic research : JCDR 2017;11(4):Td01-td02. doi: 10.7860/jcdr/2017/23269.9682

64. Srinivasan KG, Balasubramanian P, Mayilvaganan KR, Kannan UN, Bilal M. Central variant of posterior reversible encephalopathy syndrome – A rare case report. Journal of Clinical and Diagnostic Research 2017;11(4):TD01-TD02. doi: 10.7860/JCDR/2017/23269.9682

65. Seol YM, Kim DY, Kim HJ, Choi YJ. Reversible Posterior Leukoencephalopathy Syndrome after Eribulin Mesylate Chemotherapy for Breast Cancer. The breast journal 2017;23(4):487-488. doi: 10.1111/tbj.12776

66. Ramirez R, Muskula PR, Everley MP. Posterior Reversible Encephalopathy Syndrome After Orthotopic Heart Transplantation: A Case Report. The American journal of case reports 2017;18:487-490.

67. Papaginovic Leiva MM, Nicolini ME, Mojico ER, Serrano MH, Umansky M, Martinez RD. Four cases of posterior reversible encephalopathy syndrome (PRES) with acute and chronic kidney disease. Should we nephrologists consider it more frequently? Blood Purification 2017;44(3):163-165. doi: 10.1159/000477427

68. Pan D, Sabharwal B, Vallejo F. Posterior Reversible Encephalopathy Syndrome Secondary to Cyclophosphamide in the Treatment of Pulmonary Renal Syndrome. Chest 2017;152(4):367a-367a. doi: 10.1016/j.chest.2017.08.393

69. Niwa R, Oya S, Nakamura T, Hana T, Matsui T. Rapid intracranial pressure drop as a cause for posterior reversible encephalopathy syndrome: Two case reports. Surgical neurology international 2017;8:103. doi: 10.4103/sni.sni_55_17

70. Nasu K, Fujisawa M, Kato H, Nangaku M. Three cases of posterior reversible encephalopathy syndrome with chronic kidney disease triggered by infection. Nephrology (Carlton, Vic) 2017;22(4):322-325. doi: 10.1111/nep.12930

71. Meenakshi-Sundaram S, Senthilnathan S, Srinivasan KG, Karthik SN, Suresh P, Palanirajan S. Reversible posterior leukoencephalopathy syndrome: single photon emission computerized tomography observations. Neuroimmunology and Neuroinflammation 2017;4(2):28-32. doi: 10.20517/2347-8659.2016.12

72. Mastorodemos V, Ioannides S, Papadaki E, Mitsias P. Posterior reversible encephalopathy syndrome in a patient with multiple sclerosis. J Neurol Sci 2017;381:784-784. doi: 10.1016/j.jns.2017.08.2214

73. Magsi S, Zafar A. Malignant Posterior Reversible Encephalopathy Syndrome-An Exacting Challenge for Neurocritical Care Physicians. The Neurohospitalist 2017;7(4):196-199. doi: 10.1177/1941874416688989

74. Lucchesi M, Mascalchi M, Mussa F, Favre C, Genitori L, Sardi I. Spinal cord involvement in two children with posterior reversible encephalopathy syndrome. CNS oncology 2017;6(4):287-290. doi: 10.2217/cns-2017-0003

75. Lu H, Lahiri S. Posterior reversible encephalopathy syndrome presenting with cerebellar edema and obstructive hydrocephalus. Neurocritical Care 2017;27(2):S422. doi: 10.1007/s12028-017-0465-9

76. Kitano T, Nezu T, Mukai T, Uemura J, Wada Y, Yagita Y. A Case of Hypertensive Encephalopathy with Enlarged Optic Nerve Sheath Measured by Transorbital Sonography. Journal of stroke and cerebrovascular diseases : the official journal of National Stroke Association 2017;26(1):e20-e21. doi: 10.1016/j.jstrokecerebrovasdis.2016.10.014

77. Jia LJ, Qu ZZ, Zhang XQ, Tian YJ, Wang Y. Uremic encephalopathy with isolated brainstem involvement revealed by magnetic resonance image: a case report. BMC neurology 2017;17(1):154. doi: 10.1186/s12883-017-0936-9

78. Ibrahim AM, ElSefi TT, Ghanem M, Fayed AM, Shaban NA. A Horned Viper Bite Victim with PRES. Case reports in neurological medicine 2017;2017:1835796. doi: 10.1155/2017/1835796

79. Hussein HM, Dornfeld B, Schneider DJ. Nivolumab-induced posterior reversible encephalopathy syndrome. Neurology Clinical practice 2017;7(5):455-456. doi: 10.1212/cpj.0000000000000362

80. Hosseini AA, Ali F, Allroggen H. AN ATYPICAL CASE OF POSTERIOR REVERSIBLE ENCEPHALOPATHY SYNDROME. Journal of Neurology Neurosurgery and Psychiatry 2017;88:A72-A72. doi: 10.1136/jnnp-2017-ABN.250

81. Hernandez-Duran S, Barrantes-Freer A, Rohde V, von der Brelie C. Posterior reversible encephalopathy syndrome presenting in the anterior circulation with malignant intracranial hypertension requiring surgical decompression: a case report and literature review. Acta neurochirurgica 2017;159(7):1321-1324. doi: 10.1007/s00701-017-3197-x

82. Helton K, Patterson AL, Khan RB, Sadighi ZS. The Complex Diagnostic Challenge in Children With Non-Central Nervous System Cancer and Cerebellar Mutism. Journal of child neurology 2017;32(9):823-827. doi: 10.1177/0883073817709178

83. Hadjipanayis A, Efstathiou E, Theophilou L, Chrousos G. Reversible brain lesion following growth hormone replacement therapy in an adolescent. BMJ case reports 2017;2017. doi: 10.1136/bcr-2017-221885

84. Hadad LK, Billingsley CC. Posterior reversible encephalopathy syndrome (PRES) associated with ovarian cancer and voltage-gated potassium channel antibodies: A case report. Gynecologic oncology reports 2017;20:67-69. doi: 10.1016/j.gore.2017.02.010

85. Gokhale A, Kimona A, Kantor S, Prakash S, Manhas Y. Posterior Reversible Leukoencephalopathy Syndrome (PRES) in Intensive Care Unit - Case series. Indian journal of critical care medicine : peer-reviewed, official publication of Indian Society of Critical Care Medicine 2017;21(11):772-778. doi: 10.4103/ijccm.IJCCM_235_17

86. Gocmen R, Ardicli D, Erarslan Y, Duzova A, Anlar B. Reversible Hypertensive Myelopathy-The Spinal Cord Variant of Posterior Reversible Encephalopathy Syndrome. Neuropediatrics 2017;48(2):115-118. doi: 10.1055/s-0036-1597612

87. Gheith O, Cerna M, Halim MA, Nampoory N, Al-Otaibi T, Nair P, Said T, Atteya HA, Katchy K. Sirolimus-Induced Combined Posterior Reversible Encephalopathy Syndrome and Lymphocytic Pneumonitis in a Renal Transplant Recipient: Case Report and Review of the Literature. Experimental and clinical transplantation : official journal of the Middle East Society for Organ Transplantation 2017;15(Suppl 1):170-174. doi: 10.6002/ect.mesot2016.P36

88. Furubayashi N, Negishi T, Iwai H, Nagase K, Nakamura M. Sorafenib-induced reversible posterior leukoencephalopathy in patients with renal cell carcinoma: A report of two cases. Molecular and clinical oncology 2017;7(2):281-284. doi: 10.3892/mco.2017.1291

89. Frick D, Huecker M, Shoff H. Posterior Reversible Encephalopathy Syndrome Presenting as Stroke Mimic. Clinical practice and cases in emergency medicine 2017;1(3):171-174. doi: 10.5811/cpcem.2017.1.30607

90. Freedman D, Koram A, Gillson N, Aylward SC. Pediatric Posterior Reversible Encephalopathy Syndrome (PRES) With Spinal Cord Involvement Due to Pheochromocytoma. Pediatr Neurol 2017;77:92-93. doi: 10.1016/j.pediatrneurol.2017.06.016

91. Fitzgerald RT, Santoro J, Hinduja A, Samant RS, Kumar M, Angtuaco EJ. PRES and Epilepsy: A Potential Long-Term Consequence of a "Reversible" Syndrome. The neurologist 2017;22(2):41-43. doi: 10.1097/nrl.0000000000000103

92. Ferrara M, Di Viesti P, Inchingolo V, Latino RR, Popolizio T, De Cosmo SA, Pugliese F, Leone MA. Isolated pons involvement in Posterior Reversible Encephalopathy Syndrome: Case report and review of the literature. eNeurologicalSci 2017;6:51-54. doi: 10.1016/j.ensci.2016.11.008

93. Feil K, Forbrig R, Thaler FS, Conrad J, Heck S, Dorn F, Pfister HW, Straube A. Reversible cerebral vasoconstriction syndrome and posterior reversible encephalopathy syndrome associated with intracranial hypotension. Neurocrit Care 2017;26(1):103-108. doi: 10.1007/s12028-016-0320-4

94. Eroglu N, Bahadir A, Erduran E. A Case of ALL Developing Posterior Reversible Encephalopathy Secondary to Hyponatremia. Journal of pediatric hematology/oncology 2017;39(8):e476-e478. doi: 10.1097/mph.0000000000000827

95. Dos Santos D, Langer FW, Dos Santos T, Rafael Tronco Alves G, Feiten M, Teixeira de Paula Neto W. Posterior reversible encephalopathy syndrome as a complication of Henoch-Schonlein purpura in a seven-year-old girl. Scottish medical journal 2017;62(1):34-37. doi: 10.1177/0036933017690467

96. Debray S, Van de Vondel S, Tousseyn T, Thal DR, Dewil M. Posterior Reversible Encephalopathy Syndrome in a Patient With Multiple System Atrophy. Movement disorders clinical practice 2017;4(5):789-790. doi: 10.1002/mdc3.12495

97. De Angeli F, Piatti M, Santoro P, Fumagalli L, Appollonio I, Ferrarese C. Thunderclap headache PRES-related during puerperium in a patient with tension-type headache: A real case. Neurological Sciences 2017;38(1):S209-S210. doi: 10.1007/s10072-017-2951-4

98. Chen TH, Lin WC, Kao WT, Tseng CM, Tseng YH. Posterior Reversible Encephalopathy Syndrome With Spinal Cord Involvement in Children. Journal of child neurology 2017;32(1):112-119. doi: 10.1177/0883073816671237

99. Castillo A, Payne JD, Nugent K. Posterior reversible leukoencephalopathy syndrome after kratom ingestion. Proceedings (Baylor University Medical Center) 2017;30(3):355-357.

100. Bede P, El-Kininy N, O'Hara F, Menon P, Finegan E, Healy D. 'Khatatonia' - cathinone-induced hypertensive encephalopathy. The Netherlands journal of medicine 2017;75(10):448-450.

101. Bazuaye-Ekwuyasi E, Chow RD, Schmalzle S. An atypical subacute presentation of posterior reversible encephalopathy syndrome. Journal of community hospital internal medicine perspectives 2017;7(4):269-274. doi: 10.1080/20009666.2017.1369381

102. Alavi S, Ashiani AM. Diamond-blackfan anemia complicated by PRES syndrome. Iranian Journal of Blood and Cancer 2017;9(3):15.

103. Abusabha Y, Petridis AK, Kraus B, Kamp MA, Steiger HJ, Beseoglu K. Life-threatening posterior reversible encephalopathy syndrome in the cerebellum treated by posterior fossa decompression. Acta neurochirurgica 2017;159(7):1325-1328. doi: 10.1007/s00701-017-3228-7

104. Abdennadher M, Pokala K, Mouti M, Ellington K, Warach S. Isolated pontine posterior reversible encephalopathy syndrome (PRES) presenting with transient right hemiparesis. Neurology 2017;88(16).

105. Abalo-Lojo JM, Baleato-Gonzalez S, Gonzalez F. Cortical blindness secondary to posterior reversible encephalopathy syndrome, recovered by successful blood pressure management. Arquivos brasileiros de oftalmologia 2017;80(5):324-326. doi: 10.5935/0004-2749.20170079

106. Zhang YX, Zheng Y, Zhang BJ, Zhang Y, Ding MP, Zhang BR. Variant Type of Posterior Reversible Encephalopathy Syndrome with Diffuse Cerebral White Matter and Brainstem Involvement Associated with Intracranial Hemorrhage. Journal of stroke and cerebrovascular diseases : the official journal of National Stroke Association 2016;25(12):e233-e235. doi: 10.1016/j.jstrokecerebrovasdis.2016.09.033

107. Zappella N, Perier F, Pico F, Palette C, Muret A, Merceron S, Girbovan A, Marquion F, Legriel S. Duloxetine-related posterior reversible encephalopathy syndrome: A case report. Medicine 2016;95(33):e4556. doi: 10.1097/md.0000000000004556

108. Yis U, Karaoglu P, Kurul SH, Soylu A, Cakmakci H, Kavukcu S. Posterior reversible leukoencephalopathy syndrome with spinal cord involvement in a 9-year-old girl. Brain & development 2016;38(1):154-157. doi: 10.1016/j.braindev.2015.07.001

109. Yafour N, Krim A, Bouhass R, Bekadja MA. Cyclosporine-related brainstem atypical posterior reversible leukoencephalopathy syndrome following hematopoietic stem cell transplant. Hematology/oncology and stem cell therapy 2016;9(1):36-38. doi: 10.1016/j.hemonc.2015.04.005

110. Xie C, Jones VT. Reversible posterior leukoencephalopathy syndrome following combinatorial cisplatin and pemetrexed therapy for lung cancer in a normotensive patient: A case report and literature review. Oncology letters 2016;11(2):1512-1516. doi: 10.3892/ol.2015.4059

111. Tsai SJ, Yeh CB, Wang CW, Mao WC, Yeh TC, Tai YM, Lee YF, Chang HA, Kao YC, Tzeng NS. Delusional infestation in a patient with posterior reversible encephalopathy syndrome. The Australian and New Zealand journal of psychiatry 2016;50(12):1212-1213. doi: 10.1177/0004867416656259

112. Thakre M, Inshasi J. Case of fatal posterior reversible encephalopathy syndrome presenting as cerebellar edema and reverse herniation. Neurology 2016;86(16).

113. Tantikittichaikul S, Ruthirago D, Ali S, Claudio A, Kim J. Posterior reversible encephalopathy syndrome with multiple cerebellar mass-like lesions secondary to amphetamine. Neurology 2016;86(16).

114. Sigurta A, Terzi V, Regna-Gladin C, Fumagalli R. Posterior Reversible Encephalopathy Syndrome Complicating Traumatic Pancreatitis: A Pediatric Case Report. Medicine 2016;95(22):e3758. doi: 10.1097/md.0000000000003758

115. Rahmanzadeh R, Rahmanzade R, Zabihiyeganeh M. Posterior reversible encephalopathy syndrome in a patient with mixed connective tissue disease: a case report. J Med Case Rep 2016;10(1):145. doi: 10.1186/s13256-016-0955-y

116. Ract I, Poujade A, Carsin-Nicol B, Mouriaux F, Ferre JC. Spinal cord involvement in posterior reversible encephalopathy syndrome (PRES). Journal of neuroradiology Journal de neuroradiologie 2016;43(1):56-58. doi: 10.1016/j.neurad.2015.09.004

117. Parikh S, Tavri S, Mohite S. Recurarization in a successfully managed case of posterior reversible encephalopathy syndrome (PRES) for emergency caesarean section. Anesthesia, essays and researches 2016;10(2):370-372. doi: 10.4103/0259-1162.167833

118. Paolini S, Jadhav AP. Teaching NeuroImages: Posterior reversible encephalopathy syndrome resulting in hydrocephalus. Neurology 2016;86(23):e242-243. doi: 10.1212/wnl.0000000000002746

119. Ohira J, Mori N, Kajikawa S, Nakamura T, Arisato T, Takahashi M. Posterior Reversible Encephalopathy Syndrome with Extensive Deep White Matter Lesions Including the Temporal Pole. Internal medicine (Tokyo, Japan) 2016;55(23):3529-3533. doi: 10.2169/internalmedicine.55.7324

120. Nanba T, Kashimura H, Saura H, Takeda M. Subarachnoid hemorrhage due to ruptured intracranial aneurysm following posterior reversible encephalopathy syndrome. Journal of Neurosciences in Rural Practice 2016;7(3):440-442. doi: 10.4103/0976-3147.182767

121. Nakagawa H, Mizuno Y, Harada E, Morikawa Y, Kuwahara K, Saito Y, Yasue H. Brain Natriuretic Peptide Counteracting the Renin-angiotensin-aldosterone System in Accelerated Malignant Hypertension. The American journal of the medical sciences 2016;352(5):534-539. doi: 10.1016/j.amjms.2016.08.001

122. Marrone LC, Martins WA, Brunelli JP, Fussiger H, Carvalhal GF, Filho JR, Soder RB, Schuck M, Viola FS, Marrone AC, da Costa JC. PRES with asymptomatic spinal cord involvement. Is this scenario more common than we know? Spinal cord series and cases 2016;2:15001. doi: 10.1038/scsandc.2015.1

123. Mahevas T, Lidove O, Yahia SA, Hayet S, Xerri-Campano B, Ziza JM. Posterior reversible encephalopathy syndrome (PRES) in a patient taking adalimumab for spondyloarthritis. Joint, bone, spine : revue du rhumatisme 2016;83(2):243-244. doi: 10.1016/j.jbspin.2015.08.002

124. Madaelil TP, Dhar R. Posterior reversible encephalopathy syndrome with thalamic involvement during vasopressor treatment of vertebrobasilar vasospasm after subarachnoid hemorrhage. Journal of neurointerventional surgery 2016;8(11):e45. doi: 10.1136/neurintsurg-2015-012103.rep

125. Ko YJ, Lee YG, Park JW, Ahn SH, Kwak JM, Choi YH. A Comprehensive Rehabilitation Approach in a Patient With Serious Neuropsychiatric Systemic Lupus Erythematosus. Annals of rehabilitation medicine 2016;40(4):745-750. doi: 10.5535/arm.2016.40.4.745

126. Khokhar H, Choudhary P, Saxena S, Arif M. Posterior reversible encephalopathy syndrome with spinal cord involvement (PRES-SCI): A case report. Annals of Indian Academy of Neurology 2016;19(1):134-136. doi: 10.4103/0972-2327.165456

127. Katz-Agranov N, Padilla J, Scoon J, Cherian SV. Carfilzomib: a rare cause of posterior reversible encephalopathy syndrome. Annals of hematology 2016;95(11):1923-1924. doi: 10.1007/s00277-016-2769-9

128. Iwafuchi Y, Okamoto K, Oyama Y, Narita I. Posterior Reversible Encephalopathy Syndrome in a Patient with Severe Uremia without Hypertension. Internal medicine (Tokyo, Japan) 2016;55(1):63-68. doi: 10.2169/internalmedicine.55.5563

129. Ito T, Okamoto R, Taniguchi M, Tanabe M, Komatsubara H, Yamada N, Ito M. Hypertensive emergency preceding the progression of periaortitis and retroperitoneal fibrosis: case report and review of the literature. Blood pressure 2016;25(5):327-330. doi: 10.3109/07434618.2016.1168968

130. Issa G, Nasser S, Kodsi S, Farhat Z. Diffuse leukoencephalopathy in a 29-year-old male with hypertensive emergency. BJR case reports 2016;2(4):20150199. doi: 10.1259/bjrcr.20150199

131. Hong G, Kim N, Lee H, Woo JH. Management of CNI induced posterior reversible encephalopathy syndrome with everolimus change after living donor liver transplantation (case). Transplantation 2016;100(7):S538. doi: 10.1097/01.tp.0000490147.72544.1a

132. Ho T-H, Kao H-W, Chen S-J, Lee J-T, Hsu Y-D. Hypertensive brainstem encephalopathy mimicking central pontine myelinolysis: a potential pitfall. American Journal of Emergency Medicine 2016;34(9). doi: 10.1016/j.ajem.2016.02.040

133. Ho TH, Tsai CL, Hsu YD, Lee JT, Yang FC, Hsu CC, Lin CC. Posterior Reversible Encephalopathy Syndrome Mimicking Brainstem Infarction: A Dilemma. Acta neurologica Taiwanica 2016;25(2):56-59.

134. Hattori N, Yamamoto K, Kawaguchi Y, Fujiwara S, Arai N, Kabasawa N, Tsukamoto H, Uto Y, Yanagisawa K, Saito B, Nakamaki T. Early relapse of severe chronic active Epstein-Barr virus infection with posterior reversible encephalopathy syndrome after reduced intensity stem cell transplantation with umbilical cord blood. Leukemia & lymphoma 2016;57(10):2448-2451. doi: 10.3109/10428194.2016.1142081

135. Garfall AL, Lancaster E, Stadtmauer EA, Lacey SF, Dengel K, Ambrose DE, Chen F, Gupta M, Kulikovskaya I, Vogl DT, Plesa G, Weiss BM, Ferthio R, Richardson C, Melenhorst JJ, Levine BL, June CH, Milone M, Cohen AD. Posterior reversible encephalopathy syndrome (PRES) after infusion of anti-Bcma CAR T Cells (CART-BCMA) for multiple myeloma: Successful treatment with cyclophosphamide. Blood 2016;128(22).

136. Fukui S, Toyoshima Y, Inoue T, Kagebayashi Y, Samma S. Reversible Posterior Leukoencephalopathy Syndrome Developing After Restart of Sunitinib Therapy for Metastatic Renal Cell Carcinoma. Case reports in medicine 2016;2016:6852951. doi: 10.1155/2016/6852951

137. Franken TP, Demaerel P, Vandenberghe W. Posterior reversible encephalopathy syndrome in multiple system atrophy. Parkinsonism & related disorders 2016;28:155-156. doi: 10.1016/j.parkreldis.2016.03.016

138. Ferreira TS, Reis F, Appenzeller S. Posterior reversible encephalopathy syndrome and association with systemic lupus erythematosus. Lupus 2016;25(12):1369-1376. doi: 10.1177/0961203316643598

139. Ferrara M, Di Viesti MP, Inchingolo MV, Latino MR, Popolizio MT, Leone M. A case of posterior reversible encephalopathy syndrome with isolated pons involvement in a patient with Turner syndrome. European Journal of Neurology 2016;23:270. doi: 10.1111/ene.13092

140. Corea F, Maselli A, Falcinelli F, Micheli S, Lentischio L, Zava R, Stefanucci S, Zampolini M. A case of IOR reversible encephalopathy syndrome (PRES) treated with percutaneous transluminal angioplasty. European Stroke Journal 2016;1(1):294-295. doi: 10.1177/2396987316642909

141. Compagnone C, Bellantonio D, Pavan F, Tagliaferri F, Barbagallo M, Fanelli G. Posterior reversible encephalopathy syndrome in acute pancreatitis. Minerva anestesiologica 2016;82(11):1236-1237.

142. Chakroun-Walha O, Bacha I, Frikha M, Ben Mahfoudh K, Rekik N. A rare case of acute posterior reversible encephalopathy syndrome involving brainstem in a child. Journal of Acute Disease 2016;5(6):521-523. doi: 10.1016/j.joad.2016.08.026

143. Brown GD, Muzyk AJ, Preud'homme XA. Prolonged Delirium With Catatonia Following Orthotopic Liver Transplant Responsive to Memantine. Journal of psychiatric practice 2016;22(2):128-132. doi: 10.1097/pra.0000000000000133

144. Borovac JA, Božić J, Žaja N, Kolić K, Hrboka V. A global amnesia associated with the specific variant of posterior reversible encephalopathy syndrome (PRES) that developed due to severe preeclampsia and malignant hypertension. Oxford Medical Case Reports 2016;2016(4):76-80. doi: 10.1093/omcr/omw016

145. Bo Q-Y, Zhao X-H, Yang X, Wang S-J. Reversible posterior encephalopathy syndrome associated with late onset postpartum eclampsia: A case report. Experimental and Therapeutic Medicine 2016;12(3):1885-1888. doi: 10.3892/etm.2016.3533

146. Bican Demir A, Erer Ozbek S, Bora I, Hakyemez B, Tirnova I, Kaya E. Two Cases With Developing Neurologic Complications After Liver Transplant. Experimental and clinical transplantation : official journal of the Middle East Society for Organ Transplantation 2016;14(6):685-687. doi: 10.6002/ect.2014.0204

147. Barker M, Alnaggar E. Posterior reversible encephalopathy syndrome associated with postpartum atypical pre-eclampsia-a case report and literature review. Australian and New Zealand Journal of Obstetrics and Gynaecology 2016;56:31.

148. Agarwal H, Sebastian LJD, Gaikwad SB, Garg A, Mishra NK. Spinal cord involvement and contrast enhancement in posterior reversible encephalopathy syndrome. BJR case reports 2016;2(1):20150326. doi: 10.1259/bjrcr.20150326

149. Abughanimeh O, Ghanimeh MA, Qasrawi A. Posterior reversible encephalopathy syndrome (PRES) in a patient with metastatic gastric cancer while receiving FOLFIRI chemotherapy: A case report. American Journal of Gastroenterology 2016;111:S1107-S1108. doi: 10.1038/ajg.2016.375

150. Abramson M. Pres as an initial presentation of ESRD. American Journal of Kidney Diseases 2016;67(5):A19.

151. Abdullah HM, Ullah W, Ahmad E, Anwer F. Posterior reversible encephalopathy syndrome in malignant hypertension secondary to focal segmental glomerulosclerosis. BMJ case reports 2016;2016. doi: 10.1136/bcr-2016-216512

152. Zhang P, Li X, Li Y, Wang J, Zeng H, Zeng X. Reversible posterior leukoencephalopathy syndrome secondary to systemic‑onset juvenile idiopathic arthritis: A case report and review of the literature. Biomedical Reports 2015;3(1):55-58. doi: 10.3892/br.2014.380

153. Zhang L, Wang Y, Shi L, Cao J, Li Z, Wang YX. Late postpartum eclampsia complicated with posterior reversible encephalopathy syndrome: a case report and a literature review. Quantitative imaging in medicine and surgery 2015;5(6):909-916. doi: 10.3978/j.issn.2223-4292.2015.12.04

154. Yu WL, Leung T, Soo Y, Lee J, Wong KS. Thrombotic thrombocytopenic purpura with concomitant small- and large-vessel thrombosis, atypical posterior reversible encephalopathy syndrome and cerebral microbleeds. Oxf Med Case Reports 2015;2015(2):179-182. doi: 10.1093/omcr/omv001

155. Webb T, Ceccarelli D, Hargroves D, Balogun I. Hypertensive encephalopathy mimicking vasculitis with pontine oedema, cerebellar white matter lesions and multiple cerebral infarctions. International Journal of Stroke 2015;10:23. doi: 10.1111/ijs.12634-6

156. Watanabe Y, Suzuki K, Takekawa H, Numao A, Asakawa Y, Fujita H, Nakamura T, Okamura M, Hirata K. Isolated pontine involvement of posterior reversible encephalopathy syndrome with concomitant ischemic cerebral infarction. Neurology and Clinical Neuroscience 2015;3(6):234-237. doi: 10.1111/ncn3.12012

157. Tortora F, Caranci F, Belfiore MP, Manzi F, Pagliano P, Cirillo S. Brainstem variant of posterior reversible encephalopathy syndrome: A case report. The neuroradiology journal 2015;28(6):634-637. doi: 10.1177/1971400915609336

158. Topkan A, Eruyar E, Titiz AP, Karadag Y, Bilen S, Oztekin N, Ak F. Hyperbaric oxygen therapy induced posterior reversible encephalopathy syndrome accompanied by charles bonnet's syndrome. Epilepsia 2015;56:241. doi: 10.1111/epi.13241

159. Tenta M, Uchida HA, Nunoue T, Umebayashi R, Okuyama Y, Kitagawa M, Maeshima Y, Sugiyama H, Wada J. Successful treatment by mycophenolate mofetil in a patient with focal segmental glomerulosclerosis associated with posterior reversible encephalopathy syndrome. CEN case reports 2015;4(2):190-195. doi: 10.1007/s13730-014-0165-7

160. Tari Capone F, Candela S, Bozzao A, Orzi F. A new case of brainstem variant of posterior reversible encephalopathy syndrome: clinical and radiological features. Neurological sciences : official journal of the Italian Neurological Society and of the Italian Society of Clinical Neurophysiology 2015;36(7):1263-1265. doi: 10.1007/s10072-014-1999-7

161. Tang KH. Oxaliplatin-induced posterior reversible encephalopathy syndrome with isolated involvement of pons. Journal of cancer research and therapeutics 2015;11(4):1022. doi: 10.4103/0973-1482.146134

162. Sachdeva V, Garg R, Pathengay A, Chandrasekharan A, Kekunnaya R. Rapidly reversible visual loss in posterior reversible encephalopathy syndrome: An ophthalmologist's enigma. Oman journal of ophthalmology 2015;8(3):211-212. doi: 10.4103/0974-620x.169886

163. Patel SJ, Paramo J, Capitle E. A young male with systemic lupus erythematosus presenting with seizures secondary to posterior reversible encephalopathy syndrome (PRES). Journal of Allergy and Clinical Immunology 2015;135(2):AB185.

164. Orhan EK, Alpsan H, Bebek N, Pınarbaşı B, Kaymakoğlu S, Özden İ, Çoban O. Role of the tacrolimus in posterior reversible encephalopathy syndrome. Nobel Medicus 2015;11(3):80-83.

165. Ocek L, Sener U, Demirtas BS, Ozcelik MM, Oztekin O, Zorlu Y. Central-Variant Posterior Reversible Encephalopathy due to Sulfasalazine: A Case Report. Medical principles and practice : international journal of the Kuwait University, Health Science Centre 2015;24(6):578-580. doi: 10.1159/000437386

166. Nishijima H, Haga R, Suzuki C, Tomiyama M. Asymmetric posterior reversible encephalopathy syndrome due to hypertensive encephalopathy. Internal medicine (Tokyo, Japan) 2015;54(8):993-994. doi: 10.2169/internalmedicine.54.3762

167. Nielsen LH, Gron BS, Ovesen PG. Posterior reversible encephalopathy syndrome postpartum. Clin Case Rep 2015;3(4):266-270. doi: 10.1002/ccr3.218

168. Naziripour A, Jeyarajasingam A, Yadullahi M, Modhia P, Singh S. Acute respiratory failure secondary to posterior reversible encephalopathy syndrome involving brainstem. Annals of Neurology 2015;78:S68. doi: 10.1002/ana.24498

169. Naser Moghadasi A, Bozorgvari K, Arzani M. An unusual radiological presentation of posterior reversible encephalopathy syndrome. Neurologia i neurochirurgia polska 2015;49(3):203-205. doi: 10.1016/j.pjnns.2015.03.006

170. Murphy T, Al-Sharief K, Sethi V, Ranger GS. Posterior Reversible Encephalopathy Syndrome (PRES) After Acute Pancreatitis. The western journal of emergency medicine 2015;16(7):1173-1174. doi: 10.5811/westjem.2015.8.28347

171. Mishima E, Hashimoto J, Akiyama Y, Seiji K, Takase K, Abe T, Ito S. Posterior reversible encephalopathy syndrome treated with renin-angiotensin system blockade. J Neurol Sci 2015;355(1-2):219-221. doi: 10.1016/j.jns.2015.06.007

172. Maciel R, Nzwalo H, Palma R, Pizhin D, Martins A, Shamassa M. The resolution of central variant of posterior reversible encephalopathy syndrome. The Neurohospitalist 2015;5(2):91-92. doi: 10.1177/1941874414557082

173. Loens S, Conzen J, Welte GS, Scharn N, Schrader C, Weissenborn K. Reversible posterior leukoencephalopathy syndrome after withdrawal of antipsychotic medication in the context of lithium intoxication. General hospital psychiatry 2015;37(3):274.e273-275. doi: 10.1016/j.genhosppsych.2015.02.011

174. Linda H, von Heijne A. A case of posterior reversible encephalopathy syndrome associated with gilenya((R)) (fingolimod) treatment for multiple sclerosis. Frontiers in neurology 2015;6:39. doi: 10.3389/fneur.2015.00039

175. Liao PY, Lee CC, Chen CY. Hypertensive brain stem encephalopathy. The American journal of emergency medicine 2015;33(1):131.e135-137. doi: 10.1016/j.ajem.2014.06.032

176. Li D, Lian L, Zhu S. Isolated cerebellar involvement in posterior reversible encephalopathy syndrome. J Neurol Sci 2015;357(1-2):101-105. doi: 10.1016/j.jns.2015.07.004

177. Lee WJ, Yeon JY, Jo KI, Kim JS, Hong SC. Reversible Cerebral Vasoconstriction Syndrome and Posterior Reversible Encephalopathy Syndrome Presenting with Deep Intracerebral Hemorrhage in Young Women. Journal of cerebrovascular and endovascular neurosurgery 2015;17(3):239-245. doi: 10.7461/jcen.2015.17.3.239

178. Lee JJ, Cho S, Park JM, Park KI. A case of hypertensive brainstem encephalopathy presenting with severe headache and unilateral hearing loss. J Neurol Sci 2015;355(1-2):211-212. doi: 10.1016/j.jns.2015.05.040

179. Landais A, Lemonne N, Etienne-Julan M. Uncommon Posterior Reversible Encephalopathy Syndrome in a Sickle-Cell Patient. Journal of clinical neurology (Seoul, Korea) 2015;11(3):287-288. doi: 10.3988/jcn.2015.11.3.287

180. Lai S, Kuang J, Fu X, Pei J, Chen L. Pheochromocytoma complicated with posterior reversible encephalopathy syndrome and cardiomyopathy. Endocrine Reviews 2015;36.

181. Ladeira F, Caetano A, Calado S, Sá F. Infratentorial variant of posterior reversible encephalopathy syndrome complicated by bilateral ischemic stroke. European Journal of Neurology 2015;22:272. doi: 10.1111/ene.12807

182. Kunzmann J, Wolf H, Oberndorfer S. Generalised reversible encephalopathy syndrome: a variant of posterior reversible encephalopathy syndrome (PRES). BMJ case reports 2015;2015. doi: 10.1136/bcr-2015-210498

183. Klomjit S, Hosiriluck N, Laengvejkal P, Panikkath D, Nugent K. Reversible posterior leukoencephalopathy syndrome induced by atixinib. Journal of Investigative Medicine 2015;63(2):444. doi: 10.1097/JIM.0000000000000146

184. Hou X, Xu J, Chen Z, Li G, Jiang H. Posterior reversible encephalopathy syndrome with involvement of the cervical cord and medulla: a case report. Journal of clinical and diagnostic research : JCDR 2015;9(1):Cd01-02. doi: 10.7860/jcdr/2015/10756.5376

185. Erkoc SK, Kayacan U, Can A, Copluoglu HE, Tosun A. Atypical Presentation of Posterior Reversible Encephalopathy Syndrome in a Patient Diagnosed with Postpartum Gestational Hypertension. Turkish journal of anaesthesiology and reanimation 2015;43(2):119-122. doi: 10.5152/tjar.2014.88557

186. Elmalik HH, ElAzzazy S, Salem KS, Bujassoum S. A Grave Outcome of Posterior Reversible Encephalopathy Syndrome in a Patient Receiving Avastin (Bevacizumab) for Metastatic High-Grade Serous Ovarian Cancer. Case reports in oncology 2015;8(2):290-294. doi: 10.1159/000435805

187. Edvardsson B. Venlafaxine as single therapy associated with hypertensive encephalopathy. SpringerPlus 2015;4:97. doi: 10.1186/s40064-015-0883-0

188. Crona DJ, Whang YE. Posterior reversible encephalopathy syndrome induced by enzalutamide in a patient with castration-resistant prostate cancer. Investigational new drugs 2015;33(3):751-754. doi: 10.1007/s10637-014-0193-3

189. Cozzolino M, Bianchi C, Mariani G, Marchi L, Fambrini M, Mecacci F. Therapy and differential diagnosis of posterior reversible encephalopathy syndrome (PRES) during pregnancy and postpartum. Archives of gynecology and obstetrics 2015;292(6):1217-1223. doi: 10.1007/s00404-015-3800-4

190. Canney M, Kelly D, Clarkson M. Posterior reversible encephalopathy syndrome in end-stage kidney disease: not strictly posterior or reversible. American journal of nephrology 2015;41(3):177-182. doi: 10.1159/000381316

191. Camlidag I, Cho YJ, Park M, Lee SK. Atypical Unilateral Posterior Reversible Encephalopathy Syndrome Mimicking a Middle Cerebral Artery Infarction. Korean journal of radiology 2015;16(5):1104-1108. doi: 10.3348/kjr.2015.16.5.1104

192. Buyukaslan H, Lok U, Gulacti U, Sogut O, Kaya H, Gokdemir T, Yalin O. Posterior reversible encephalopathy syndrome during the peripartum period: report of four cases and review of the literature. Int J Clin Exp Med 2015;8(2):1575-1581.

193. Budhoo A, Mody GM. The spectrum of posterior reversible encephalopathy in systemic lupus erythematosus. Clinical rheumatology 2015;34(12):2127-2134. doi: 10.1007/s10067-015-3055-2

194. Balasa R, Maier S, Baubec EG, Bajko Z, Balasa A. Cerebellar and brainstem variant of posterior reversible encephalopathy syndrome. Acta neurologica Belgica 2015;115(3):401-403. doi: 10.1007/s13760-014-0370-3

195. Baek HS, Lee SJ. A case of posterior reversible encephalopathy syndrome associated with acute pancreatitis and chronic alcoholism. General hospital psychiatry 2015;37(2):192.e193-195. doi: 10.1016/j.genhosppsych.2014.12.004

196. Ates S, Kemerdere R, Sevket O, Molla T, Dane B. Posterior reversible encephalopathy associated with HELLP syndrome: A case report and review of the literature. Neurol Asia 2015;20(1):91-94.

197. Arslan ZI, Turna CK, Ozerdem CY, Yavuz S, Baykara N, Solak M. Treatment of Posterior Reversible Encephalopathy Syndrome that Occurred in a Patient with Systemic Lupus Erythematosus by Plasmapheresis. Turkish journal of anaesthesiology and reanimation 2015;43(4):291-294. doi: 10.5152/tjar.2015.13540

198. Akıncı E, Köylü R, Yortanlı M, Gümüş H, Köylü Ö, Altıntepe L, Cander B. Acute bismuth intoxication: Acute renal failure, tonsillar ulceration and posterior reversible encephalopathy syndrome. Hong Kong Journal of Emergency Medicine 2015;22(2):121-125. doi: 10.1177/102490791502200208

199. Akazawa Y, Inaba Y, Hachiya A, Motoki N, Matsuzaki S, Minatoya K, Morisaki T, Morisaki H, Kosaki K, Kosho T, Koike K. Reversible cerebral vasoconstriction syndrome and posterior reversible encephalopathy syndrome in a boy with Loeys-Dietz syndrome. American journal of medical genetics Part A 2015;167a(10):2435-2439. doi: 10.1002/ajmg.a.37202

200. Ural UM, Balik G, Senturk S, Ustuner I, Cobanoglu U, Sahin FK. Posterior Reversible Encephalopathy Syndrome in a Postpartum Preeclamptic Woman without Seizure. Case reports in obstetrics and gynecology 2014;2014:657903. doi: 10.1155/2014/657903

201. Thakral A, Malhotra R, Gupta A, Khanal M, Kumar S, Hiralal, Prasad N. An unusual case of posterior reversible encephalopathy syndrome in post-renal transplant recipient. Indian Journal of Transplantation 2014;8(3):100-103. doi: 10.1016/j.ijt.2014.10.002

202. Te Riele MG, Verrips A. Severe hypomagnesaemia causing reversible cerebellopathy. Cerebellum (London, England) 2014;13(5):659-662. doi: 10.1007/s12311-014-0567-2

203. Tan JL, McClure J, Hennington L, Padiglione A, Cleland H, Ahn TB, Fedi M. The heat is on: a case of hyperthermia-induced posterior reversible encephalopathy syndrome (PRES). Neurological sciences : official journal of the Italian Neurological Society and of the Italian Society of Clinical Neurophysiology 2014;35(1):127-130. doi: 10.1007/s10072-013-1525-3

204. Stem MS, Fahim A, Trobe JD, Parmar HA, Ibrahim M. Lateral geniculate lesions causing reversible blindness in a pre-eclamptic patient with a variant of posterior reversible encephalopathy syndrome. Journal of neuro-ophthalmology : the official journal of the North American Neuro-Ophthalmology Society 2014;34(4):372-376. doi: 10.1097/wno.0000000000000120

205. Soomro A, Al Bahri R, Alhassan N, Hejaili FF, Al Sayyari AA. Posterior reversible encephalopathy syndrome with tactile hallucinations secondary to dialysis disequilibrium syndrome. Saudi journal of kidney diseases and transplantation : an official publication of the Saudi Center for Organ Transplantation, Saudi Arabia 2014;25(3):625-629.

206. Sharma S, Gupta R, Sehgal R, Aggarwal KC. Atypical presentation of posterior reversible encephalopathy: in a child with bilateral grade IV vesicoureteric reflux. Journal of tropical pediatrics 2014;60(4):331-333. doi: 10.1093/tropej/fmu019

207. Sengupta S, Benkers T, Blitstein M, Palmer E, Plotkin SR, Abramson JS. Posterior reversible encephalopathy syndrome (PRES) complicating newly-diagnosed diffuse large B-cell lymphoma. Clinical lymphoma, myeloma & leukemia 2014;14(4):e111-113. doi: 10.1016/j.clml.2014.01.005

208. Sato S, Nakajima J, Shimura M, Kawashima H, Yoshio T, Hara Y. Reversible basal ganglia lesions in neuropsychiatric lupus: a report of three pediatric cases. International journal of rheumatic diseases 2014;17(3):274-279. doi: 10.1111/1756-185x.12235

209. Sajan T, Vinay S, Sonu N, Alan P. How atypical can Atypical Hemolytic Uremic Syndrome be? Clin Case Rep 2014;2(2):57-59. doi: 10.1002/ccr3.59

210. Roy S, Gandhi AK, Jana M, Julka PK. Recurrent posterior reversible encephalopathy syndrome after chemotherapy in hematologic malignancy-posterior reversible encephalopathy syndrome can strike twice!!! Journal of cancer research and therapeutics 2014;10(2):393-396. doi: 10.4103/0973-1482.136668

211. Ramanathan RS, Malhotra K, Guduru Z, Shaikh N, Rana S. Conversion of Posterior reversible encephalopathy syndrome into irreversible cytotoxic edema and acute infarct. Journal of Neuroimaging 2014;24(3):318-319. doi: 10.1111/jon.12109

212. Poma S, Delmonte MP, Gigliuto C, Imberti R, Delmonte M, Arossa A, Iotti GA. Management of posterior reversible syndrome in preeclamptic women. Case reports in obstetrics and gynecology 2014;2014:928079. doi: 10.1155/2014/928079

213. Patel UV, Patel NJ. Posterior reversible leukoencephalopathy syndrome as a presenting manifestation of p-ANCA-associated vasculitis. BMJ case reports 2014;2014. doi: 10.1136/bcr-2013-202022

214. Ozkan E, Gocmen R, Topcuoglu MA, Arsava EM. Blood-retina-barrier disruption accompanying blood-brain-barrier dysfunction in posterior reversible encephalopathy syndrome. J Neurol Sci 2014;346(1-2):315-317. doi: 10.1016/j.jns.2014.08.035

215. Nixon NA, Parhar K. Posterior reversible encephalopathy syndrome resulting from repeat bortezomib usage. BMJ case reports 2014;2014. doi: 10.1136/bcr-2014-204592

216. Myint ZW, Sen JM, Watts NL, Druzgal TJ, Nathan BR, Ward MD, Boyer JE, Fracasso PM. Reversible posterior leukoencephalopathy syndrome during regorafenib treatment: a case report and literature review of reversible posterior leukoencephalopathy syndrome associated with multikinase inhibitors. Clinical colorectal cancer 2014;13(2):127-130. doi: 10.1016/j.clcc.2013.12.003

217. Moris D, Vernadakis S, Lionaki S, Daikos G, Zavos G. An uncommon cause of acutely altered mental status in a renal transplant recipient. Upsala journal of medical sciences 2014;119(1):50-54. doi: 10.3109/03009734.2013.842618

218. Min Z, Gnann JW, Jr. Intern Emerg Med 2014;9(3):341-342. doi: 10.1007/s11739-013-1011-1

219. Milani GP, Edefonti A, Tardini G, Arturi E, Cinnante CM, Laicini EA, Leva E, Cappellari AM, Agostoni C, Fossali EF. Severe and isolated headache associated with hypertension as unique clinical presentation of posterior reversible encephalopathy syndrome. BMC pediatrics 2014;14:190. doi: 10.1186/1471-2431-14-190

220. Matsubara Y, Manabe Y, Hattori M, Nakano Y, Takahashi Y, Narai H, Ota K, Abe K. Serial MR spectroscopy in hypertensive encephalopathy. J Neurol Sci 2014;347(1-2):393-395. doi: 10.1016/j.jns.2014.10.004

221. Maria Alexandre A, Napoli G, Sanna A, Leoni C, Ruggiero M. Posterior reversible encephalopathy syndrome in children, epiphenomenon of a known or unknown disease. Journal of Pediatric Neuroradiology 2014;3(2):99-103. doi: 10.3233/PNR-14095

222. Mackay DD, Zepeda Garcia R, Galetta SL, Prasad S. Periodic alternating gaze deviation and nystagmus in posterior reversible encephalopathy syndrome. Neurology Clinical practice 2014;4(6):482-485. doi: 10.1212/cpj.0000000000000056

223. Larmour K, Lewis G, Benson G, Hanko J. The challenges of antiphospholipid syndrome: experience from diagnosis to self-care. BMJ case reports 2014;2014. doi: 10.1136/bcr-2014-205072

224. Kim CA, Price-Hiller J, Chu QS, Tankel K, Hennig R, Sawyer MB, Spratlin JL. Atypical reversible posterior leukoencephalopathy syndrome (RPLS) induced by cediranib in a patient with metastatic rectal cancer. Investigational new drugs 2014;32(5):1036-1045. doi: 10.1007/s10637-014-0113-6

225. Joos ZP, Adesina OO, Katz BJ. Posterior ischemic optic neuropathy in the setting of posterior reversible encephalopathy syndrome and hypertensive emergency. Journal of neuro-ophthalmology : the official journal of the North American Neuro-Ophthalmology Society 2014;34(2):151-152. doi: 10.1097/wno.0000000000000108

226. Honca M, Polat A, Horasanli E. Posterior Reversible Encephalopathy Syndrome in an Eclamptic Patient After Cardiac Arrest; Case Report and Literature Review. Turkish journal of anaesthesiology and reanimation 2014;42(1):50-53. doi: 10.5152/tjar.2014.26817

227. Hayes D, Jr., Adler B, Turner TL, Mansour HM. Alternative tacrolimus and sirolimus regimen associated with rapid resolution of posterior reversible encephalopathy syndrome after lung transplantation. Pediatr Neurol 2014;50(3):272-275. doi: 10.1016/j.pediatrneurol.2013.11.006

228. Guler T, Cakmak OY, Toprak SK, Kibaroglu S, Can U. Intrathecal Methotrexate-Induced Posterior Reversible Encephalopathy Syndrome (PRES). Turkish journal of haematology : official journal of Turkish Society of Haematology 2014;31(1):109-110. doi: 10.4274/Tjh.2012.0191

229. Guirola R, Hunter JV, Perez M, Muscal E. Childhood polyarteritis nodosa presenting with central nervous system manifestations and the posterior reversible encephalopathy syndrome. Journal of child neurology 2014;29(1):103-107. doi: 10.1177/0883073812465339

230. Grelat M, Debaux J-B, Sautreaux J-L. Posterior reversible encephalopathy syndrome after depletive lumbar puncture: a case report. Journal of medical case reports 2014;8:261-261. doi: 10.1186/1752-1947-8-261

231. Glasgo B, Bhatt N, Reilly S. Posterior reversible encephalopathy syndrome associated with hemorrhagic fever with renal syndrome from dobrava virus infection. Chest 2014;146(4). doi: 10.1378/chest.1992382

232. Geevasinga N, Cole C, Herkes GK, Barnett Y, Lin J, Needham M. Sickle cell disease and posterior reversible leukoencephalopathy. Journal of clinical neuroscience : official journal of the Neurosurgical Society of Australasia 2014;21(8):1329-1332. doi: 10.1016/j.jocn.2013.10.028

233. Freitas T, Greco R, Morelli M, Levati G, Giglio F, Assanelli A, Peccatori J, Marktel S, Ciceri F. Posterior reversible encephalopathy syndrome (PRES) associated to sirolimus administration: A case report. Bone Marrow Transplantation 2014;49:S479. doi: 10.1038/bmt.2014.50

234. Forouzanfar M, Haroutunian P, Baratloo A, Hashemi B. Posterior reversible encephalopathy syndrome as the first presentation of chronic kidney disease. The American journal of emergency medicine 2014;32(5):489.e481-483. doi: 10.1016/j.ajem.2013.11.013

235. Ettinger N, Pearson M, Lamb FS, Wellons JC, 3rd. Pediatric posterior reversible encephalopathy syndrome presenting with isolated cerebellar edema and obstructive hydrocephalus. Journal of neurosurgery Pediatrics 2014;14(4):344-347. doi: 10.3171/2014.6.Peds13553

236. de Havenon A, Joos Z, Longenecker L, Shah L, Ansari S, Digre K. Posterior reversible encephalopathy syndrome with spinal cord involvement. Neurology 2014;83(22):2002-2006. doi: 10.1212/wnl.0000000000001026

237. Coenen F, Duprez T, Hantson P. Concomitant occurrence of posterior reversible encephalopathy syndrome and non-convexal subarachnoid haemorrhage in a renal transplant patient. Acta neurologica Belgica 2014;114(2):159-161. doi: 10.1007/s13760-013-0259-6

238. Chen DY, Tseng YC, Hsu HL, Huang YL, Chen CJ. Teaching neuroimages: Central variant of posterior reversible encephalopathy syndrome. Neurology 2014;82(19):e164. doi: 10.1212/wnl.0000000000000407

239. Barnaure I, Horvath J, Lovblad KO, Vargas MI. Atypical brainstem presentation of posterior reversible encephalopathy syndrome (PRES). Journal of neuroradiology Journal de neuroradiologie 2014;41(2):143-144. doi: 10.1016/j.neurad.2013.05.002

240. Arimura FE, Camargo PCLB, Costa AN, Teixeira RHOB, Carraro RM, Afonso JE, Campos SV, Samano MN, Fernandes LM, Abdalla LG, Pêgo-Fernandes PM. Posterior reversible encephalopathy syndrome in lung transplantation: 5 case reports. Transplantation Proceedings 2014;46(6):1845-1848. doi: 10.1016/j.transproceed.2014.05.032

241. Akin F, Kilicaslan C, Solak ES, Uzun M, Aygun S, Arslan S. Posterior reversible encephalopathy syndrome in children: report of three cases. Child's nervous system : ChNS : official journal of the International Society for Pediatric Neurosurgery 2014;30(3):535-540. doi: 10.1007/s00381-013-2342-y

242. Abe T, Tokuda Y. Recurrent posterior reversible encephalopathy syndrome of the brainstem in a hypertensive patient with end-stage renal disease. Journal of emergencies, trauma, and shock 2014;7(3):242-243. doi: 10.4103/0974-2700.136876

243. Yilmaz H, Mavioglu H, Kisabay A, Oktan B. Posterior reversible encephalopathy syndrome (PRES): A case report. Epilepsia 2013;54:129. doi: 10.1111/epi.12229

244. Sivakumar S, Rayes M, Mohamed W, Norris G. Reversible brain stem hypertensive encephalopathy presenting with obstructive hydrocephalus: Report of two cases. Neurocritical Care 2013;19(1):S315. doi: 10.1007/s12028-013-9895-1

245. Shimizu Y, Tha KK, Iguchi A, Cho Y, Yoshida A, Fujima N, Tsukahara A, Shirato H, Terae S. Isolated posterior fossa involvement in posterior reversible encephalopathy syndrome. The neuroradiology journal 2013;26(5):514-519. doi: 10.1177/197140091302600504

246. Serter A, Alkan A, Aralasmak A, Kocakoc E. Severe posterior reversible encephalopathy in pheochromocytoma: importance of susceptibility-weighted MRI. Korean journal of radiology 2013;14(5):849-853. doi: 10.3348/kjr.2013.14.5.849

247. Schusse CM, Peterson AL, Caplan JP. Posterior reversible encephalopathy syndrome. Psychosomatics 2013;54(3):205-211. doi: 10.1016/j.psym.2013.01.014

248. Ribeiro S, Monteiro M, Moreira B, Franca M. Rare posterior reversible encephalopathy syndrome in a patient with HIV. BMJ case reports 2013;2013. doi: 10.1136/bcr-2013-201495

249. Riaz N, Behnia MM, Catalano PW, Davis J. A patient with moderate post-operative hypertension presenting with posterior reversible encephalopathy syndrome: a case report. Tanaffos 2013;12(3):58-61.

250. Raj S, Killinger J, Overby P. Blood transfusion in sickle cell disease leading to posterior reversible encephalopathy syndrome (PRES). Journal of child neurology 2013;28(10):1284-1286. doi: 10.1177/0883073812453497

251. Poursadeghfard M, Raeyat M, Karamimagham S. Typical and atypical features in posterior reversible encephalopathy syndrome (PRES). Shiraz E Medical Journal 2013;14(3).

252. Pamplona J, Braz A, Cac¸ador N, Diogo M, Jacinto J, Martins C, Patricio M, Baptista T, Fragata I, Reis J. None so posterior pres. Neuroradiology 2013;55(12):1480. doi: 10.1007/s00234-013-1287-x

253. Osman Y, Imam YZ, Salem K, Al-Hail H, Uthman B, Deleu D. Isolated brainstem involvement in a patient with hypertensive encephalopathy. Case reports in neurological medicine 2013;2013:540947. doi: 10.1155/2013/540947

254. Oshikawa G, Kojima A, Doki N, Kobayashi T, Kakihana K, Tsuda H, Endo I, Kamata N, Ohashi K, Sakamaki H. Bortezomib-induced posterior reversible encephalopathy syndrome in a patient with newly diagnosed multiple myeloma. Internal medicine (Tokyo, Japan) 2013;52(1):111-114.

255. Nakajima A, Ueno Y, Shimura H, Kambe T, Nishioka K, Hattori N, Urabe T. Acute transient freezing of gait in a patient with posterior reversible encephalopathy syndrome. BMC neurology 2013;13:79. doi: 10.1186/1471-2377-13-79

256. Nagaoka Y, Ishikura K, Hamada R, Miyagawa T, Kono T, Sakai T, Hamasaki Y, Hataya H, Honda M. Severe posterior reversible encephalopathy syndrome resolved with craniectomy. Pediatrics international : official journal of the Japan Pediatric Society 2013;55(5):644-646. doi: 10.1111/ped.12084

257. Mrelashvili A, Watson RE, Wong-Kisiel LC. Posterior reversible encephalopathy syndrome in an infant. Pediatr Neurol 2013;49(5):387-388. doi: 10.1016/j.pediatrneurol.2013.06.020

258. Mechtouff L, Piegay F, Traclet J, Philit F, Boissonnat P, Hermier M, Durieu I, Cho TH, Nighoghossian N, Mornex JF. Tacrolimus-related cerebral microbleeds after lung transplantation. Case reports in transplantation 2013;2013:708961. doi: 10.1155/2013/708961

259. McKinney AM, Jagadeesan BD, Truwit CL. Central-variant posterior reversible encephalopathy syndrome: brainstem or basal ganglia involvement lacking cortical or subcortical cerebral edema. AJR American journal of roentgenology 2013;201(3):631-638. doi: 10.2214/ajr.12.9677

260. Maggi G, Lombana VA, Marcos EA, Ruiz Huerta AD, Arevalo EG, Rodriguez FG. Posterior leukoencephalopathy syndrome: Postpartum focal neurologic deficits: A report of three cases and review of the literature. Saudi journal of anaesthesia 2013;7(2):205-209. doi: 10.4103/1658-354x.114056

261. Liang H, Li D, Xu Z, Luo B. Isolated pons variant of posterior reversible encephalopathy syndrome complicated with ischemic stroke in a young patient. Neurological sciences : official journal of the Italian Neurological Society and of the Italian Society of Clinical Neurophysiology 2013;34(4):585-587. doi: 10.1007/s10072-012-1082-1

262. Li Y, Castaldo J, Bemporad J, Yacoub HA. Reversible confluent deep white matter abnormalities: a new variant of posterior reversible encephalopathy syndrome. Case reports in neurological medicine 2013;2013:536978. doi: 10.1155/2013/536978

263. Li Q, Lv F, Wei Y, Yan B, Xie P. Posterior reversible encephalopathy syndrome in a patient with systemic lupus erythematosus after cessation of oral prednisone. Neurological sciences : official journal of the Italian Neurological Society and of the Italian Society of Clinical Neurophysiology 2013;34(12):2241-2242. doi: 10.1007/s10072-013-1479-5

264. Lee VH, Temes RE, John S, Conners JJ, Bleck T, Prabhakaran S. Posterior Reversible Leukoencephalopathy Syndrome Presenting with Global Cerebral Edema and Herniation. Neurocritical Care 2013;18(1):81-83. doi: 10.1007/s12028-012-9798-6

265. Lee S, Cho BK, Kim H. Hypertensive encephalopathy with reversible brainstem edema. Journal of Korean Neurosurgical Society 2013;54(2):139-141. doi: 10.3340/jkns.2013.54.2.139

266. Labidi M, Dubé M, Brisson M, Savard M. Brainstem reversible hypertensive encephalopathy: A case report. Canadian Journal of Neurological Sciences 2013;40(3):S45-S46.

267. Kolovou V, Zampakis P, Ginopoulou A, Varvarigou A, Kaleyias J. Reversible posterior leukoencephalopathy syndrome after blood transfusion in a pediatric patient with sickle cell disease. Pediatr Neurol 2013;49(3):213-217. doi: 10.1016/j.pediatrneurol.2013.04.024

268. Khanal P, Awan F, Nguyen V. Etoposide-induced posterior reversible encephalopathy syndrome. Annals of hematology 2013;92(4):561-562. doi: 10.1007/s00277-012-1601-4

269. Kaleem ZU, Alawi A, Kumar A. Reversible obstructive hydrocephalus from hypertensive encephalopathy (ROHH). Neurocritical Care 2013;19(1):S310. doi: 10.1007/s12028-013-9895-1

270. Jung SM, Moon SJ, Kwok SK, Ju JH, Park KS, Park SH, Kim HY. Posterior reversible encephalopathy syndrome in Korean patients with systemic lupus erythematosus: risk factors and clinical outcome. Lupus 2013;22(9):885-891. doi: 10.1177/0961203313496341

271. Joa KL, Shin YI, Suh H, Kim SY, Chang JH, Ko HY. Autonomic dysreflexia-induced reversible posterior leukoencephalopathy syndrome in patients with spinal cord injury: two case reports. The journal of spinal cord medicine 2013;36(3):250-253. doi: 10.1179/2045772312y.0000000075

272. Jinnur P, Vasudevan V, Vanam V, Jinnur S, Abbas Q, Arjomand F, Dakhel M. Reversible posterior leukoencephalopathy syndrome (RPLS) in a young male with uncontrolled hypertension. Chest 2013;144(4). doi: 10.1378/chest.1704604

273. Gatla N, Annapureddy N, Sequeira W, Jolly M. Posterior reversible encephalopathy syndrome in systemic lupus erythematosus. Journal of clinical rheumatology : practical reports on rheumatic & musculoskeletal diseases 2013;19(6):334-340. doi: 10.1097/RHU.0b013e3182a21ffd

274. Fitzgerald RT, Osorio J, Panigrahy A, Mazariegos GV, Zuccoli G. Isolated leptomeningeal enhancement in tacrolimus-associated posterior reversible encephalopathy syndrome. Pediatr Neurol 2013;48(1):76-78. doi: 10.1016/j.pediatrneurol.2012.09.002

275. Dhakate N, Tandon S, Nityanand S. Posterior reversible encephalopathy syndrome in a child with acute lymphoblastic leukemia. Indian Journal of Hematology and Blood Transfusion 2013;29(4):300. doi: 10.1007/s12288-013-0302-z

276. Ciftci ED, Kibaroglu S, Benli US. Posterior reversible encephalopathy syndrome associated with tacrolimus and renal failure after liver transplantation. J Neurol Sci 2013;333:e672. doi: 10.1016/j.jns.2013.07.2324

277. Chennareddy S, Adapa R, Kishore BK, Rajasekhar L. Posterior reversible encephalopathy syndrome in systemic lupus erythematosus following methylprednisolone: report of two cases. International journal of rheumatic diseases 2013;16(6):786-788. doi: 10.1111/1756-185x.12148

278. Chen TH, Chiou SS, Lin WC, Wang IF, Chen IC, Dai ZK, Wu JR, Hsu JH. Posterior reversible encephalopathy syndrome in critically ill children: a case series. Intensive care medicine 2013;39(1):155-156. doi: 10.1007/s00134-012-2705-y

279. Avecillas-Chasin JM, Gomez G, Jorquera M, Alvarado LR, Barcia JA. Delayed posterior reversible encephalopathy syndrome (PRES) after posterior fossa surgery. Acta neurochirurgica 2013;155(6):1045-1047. doi: 10.1007/s00701-013-1690-4

280. Antony R, Al-Rahawan M, Fernandez K. Diverse manifestations of pres in hematology/oncology patients. Pediatric Blood and Cancer 2013;60:S21-S22. doi: 10.1002/pbc.24509

281. Ali WH. Ciprofloxacin-associated posterior reversible encephalopathy. BMJ case reports 2013;2013. doi: 10.1136/bcr-2013-008636

282. Alexander S, David VG, Varughese S, Tamilarasi V, Jacob CK. Posterior reversible encephalopathy syndrome in a renal allograft recipient: A complication of immunosuppression? Indian journal of nephrology 2013;23(2):137-139. doi: 10.4103/0971-4065.109439

283. Abbas O, Shamseddin A, Temraz S, Haydar A. Posterior reversible encephalopathy syndrome after bevacizumab therapy in a normotensive patient. BMJ case reports 2013;2013. doi: 10.1136/bcr-2012-007995

284. Tsutsumi M, Akimoto J, Nakajima N, Hashimoto R, Haraoka J. Two cases of posterior reversible encephalopathy syndrome resembling brainstem glioma. Clin Neurol Neurosurg 2012;114(7):1062-1065. doi: 10.1016/j.clineuro.2012.02.027

285. Tsukamoto S, Takeuchi M, Kawajiri C, Tanaka S, Nagao Y, Sugita Y, Yamazaki A, Kawaguchi T, Muto T, Sakai S, Takeda Y, Ohwada C, Sakaida E, Shimizu N, Yokote K, Iseki T, Nakaseko C. Posterior reversible encephalopathy syndrome in an adult patient with acute lymphoblastic leukemia after remission induction chemotherapy. International journal of hematology 2012;95(2):204-208. doi: 10.1007/s12185-011-0982-9

286. Swarnalatha G, Ram R, Pai BH, Dakshinamurty KV. Posterior reversible encephalopathy syndrome in minimal change disease. Indian journal of nephrology 2012;22(2):153-154. doi: 10.4103/0971-4065.97153

287. Stubgen JP. Posterior reversible encephalopathy syndrome (PRES) after granulocyte-colony stimulating factor (G-CSF) therapy: a report of 2 cases. J Neurol Sci 2012;321(1-2):35-38. doi: 10.1016/j.jns.2012.07.028

288. Seneviratna S, Ellepola H, Naik S, Haran M. Posterior reversible encephalopathy syndrome (PRES) in a young postpartum woman. BJOG: An International Journal of Obstetrics and Gynaecology 2012;119:71-72. doi: 10.1111/j.1471-0528.2012.03376.x

289. Sekine T, Ikeda K, Hirayama T, Suzuki A, Iwasaki Y. Transient splenial lesion after recovery of cerebral vasoconstriction and posterior reversible encephalopathy syndrome: a case report of eclampsia. Internal medicine (Tokyo, Japan) 2012;51(11):1407-1411.

290. Seet RCS, Rabinstein AA. Clinical features and outcomes of posterior reversible encephalopathy syndrome following bevacizumab treatment. Qjm-an International Journal of Medicine 2012;105(1):69-75. doi: 10.1093/qjmed/hcr139

291. Rosso L, Nosotti M, Mendogni P, Palleschi A, Tosi D, Montoli M, Pappalettera M, Tarsia P, Santambrogio L. Lung transplantation and posterior reversible encephalopathy syndrome: a case series. Transplant Proc 2012;44(7):2022-2025. doi: 10.1016/j.transproceed.2012.06.027

292. Parmentier C, Vandermeeren Y, Laloux P, Mormont E. Asymptomatic posterior reversible encephalopathy revealed by brain MRI in a case of axonal Guillain-Barre syndrome. Clinical Neurology and Neurosurgery 2012;114(7):1006-1009. doi: 10.1016/j.clineuro.2012.01.012

293. Ogunneye O, Hernandez-Montfort JA, Ogunneye Y, Ogu I, Landry D. Parainfluenza virus infection associated with posterior reversible encephalopathy syndrome: a case report. J Med Case Rep 2012;6:89. doi: 10.1186/1752-1947-6-89

294. Monteiro C, Almeida I, Dias D, Santos E. Atypical posterior reversible encephalopathy syndrome: a flare of systemic lupus erythematosus. The Journal of rheumatology 2012;39(1):195-196. doi: 10.3899/jrheum.110838

295. Maur M, Tomasello C, Frassoldati A, Dieci MV, Barbieri E, Conte P. Posterior reversible encephalopathy syndrome during ipilimumab therapy for malignant melanoma. Journal of clinical oncology : official journal of the American Society of Clinical Oncology 2012;30(6):e76-78. doi: 10.1200/jco.2011.38.7886

296. Lunardi N, Saraceni E, Boccagni P, Segato M, Bortolato A, Manara R, Rossi S, Ori C. Posterior reversible encephalopathy syndrome in the Intensive Care Unit after liver transplant: a comparison of our experience with the existing literature. Minerva anestesiologica 2012;78(7):847-850.

297. Liu B, Zhang X, Zhang FC, Yao Y, Zhou RZ, Xin MM, Wang LQ. Posterior reversible encephalopathy syndrome could be an underestimated variant of "reversible neurological deficits" in Systemic Lupus Erythematosus. BMC neurology 2012;12:152. doi: 10.1186/1471-2377-12-152

298. Lemmens R, Smet S, Wilms G, Demaerel P, Thijs V. Postpartum RCVS and PRES with normal initial imaging findings. Acta neurologica Belgica 2012;112(2):189-192. doi: 10.1007/s13760-012-0051-z

299. Lazarus M, Amundson S, Belani R. An Association between Bevacizumab and Recurrent Posterior Reversible Encephalopathy Syndrome in a Patient Presenting with Deep Vein Thrombosis: A Case Report and Review of the Literature. Case reports in oncological medicine 2012;2012:819546. doi: 10.1155/2012/819546

300. Lahmer T, Kuchle C, Schirmer L, Heemann U, Lutz J, Thurmel K. Kidney transplant after preexisting posterior reversible encephalopathy syndrome induced by Goodpasture's syndrome. Experimental and clinical transplantation : official journal of the Middle East Society for Organ Transplantation 2012;10(3):299-301.

301. Kumar A, Keyrouz SG, Willie JT, Dhar R. Reversible obstructive hydrocephalus from hypertensive encephalopathy. Neurocrit Care 2012;16(3):433-439. doi: 10.1007/s12028-011-9663-z

302. Komur M, Delibas A, Arslankoylu AE, Okuyaz C, Kara E. Recurrent and atypical posterior reversible encephalopathy syndrome in a child with hypertension. Ann Indian Acad Neurol 2012;15(3):208-210. doi: 10.4103/0972-2327.99721

303. Kawamura Y, Ohashi M, Asahito H, Takahashi Y, Kojima S, Yoshikawa T. Posterior reversible encephalopathy syndrome in a child with post-transplant HHV-6B encephalitis. Bone Marrow Transplant 2012;47(10):1381-1382. doi: 10.1038/bmt.2012.42

304. Kamezaki M, Kakimoto T, Takeuchi T, Akuta K, Kasahara H, Yamamoto K, Ujiie H, Sugahara H, Nishinaka K, Udaka F, Sakoda H. Reversible posterior leukoencephalopathy syndrome of bilateral thalamus in acute lymphoblastic leukemia. Leukemia & lymphoma 2012;53(10):2083-2084. doi: 10.3109/10428194.2012.673227

305. Kadkol R, Godbole RR. Antepartum eclampsia with posterior reversible encephalopathy syndrome. Journal of obstetrics and gynaecology of India 2012;62(Suppl 1):27-28. doi: 10.1007/s13224-013-0373-7

306. Ince V, Aydin C, Karakas S, Gedik E, Yilmaz S. Posterior reversible encephalopathy syndrome after living donor liver transplantation. Liver Transplantation 2012;18:S180-S181. doi: 10.1002/lt.23435

307. Guerriero S, Ciraci L, Centoducati T, Pignatelli F, Lamargese V, Salvati A, Dicuonzo F. Bilateral Visual Loss as Presenting Symptom of Posterior Reversible Encephalopathy Syndrome in a Patient with HIV/Tuberculosis Coinfection: A Case Report. Case reports in ophthalmological medicine 2012;2012:850176. doi: 10.1155/2012/850176

308. Gao B, Liang H, Liu FL, Lv C. Isolated pons involvement in posterior reversible encephalopathy syndrome in a patient with chronic renal insufficiency: case report and literature review. Clinical neuroradiology 2012;22(4):341-344. doi: 10.1007/s00062-012-0162-1

309. Dham B, Moghal U, Velazquez Y, Kavi T, Zayas L, Umer A. A fulminant case of atypical posterior reversible encephalopathy syndrome associated with status epilepticus. Annals of Neurology 2012;72:S5. doi: 10.1002/ana.23769

310. Demirel I, Ozer AB, Bayar MK, Kavak SB. Anesthesia and Intensive Care Management in a Pregnant Woman with PRES: A Case Report. Case reports in anesthesiology 2012;2012:745939. doi: 10.1155/2012/745939

311. Deguchi I, Uchino A, Suzuki H, Tanahashi N. Malignant hypertension with reversible brainstem hypertensive encephalopathy and thrombotic microangiopathy. Journal of stroke and cerebrovascular diseases : the official journal of National Stroke Association 2012;21(8):915.e917-920. doi: 10.1016/j.jstrokecerebrovasdis.2012.02.005

312. Cioffi P, Laudadio L, Nuzzo A, Belfiglio M, Petrelli F, Grappasonni I. Gemcitabine-induced posterior reversible encephalopathy syndrome: a case report. Journal of oncology pharmacy practice : official publication of the International Society of Oncology Pharmacy Practitioners 2012;18(2):299-302. doi: 10.1177/1078155211424628

313. Chen MH, Lee HF, Chang WC, Lin MC, Fu YC, Jan SL. Hypertensive encephalopathy caused by fibromuscular dysplasia of renal arteries in a child. Acta Cardiologica Sinica 2012;28(1):60-62.

314. Caputo ND, Fraser RM, Abdulkarim J. Posterior reversible encephalopathy syndrome presenting as papilledema. The American journal of emergency medicine 2012;30(5):835.e835-837. doi: 10.1016/j.ajem.2011.03.016

315. Boulos MI, Shoamanesh A, Aviv RI, Gladstone DJ, Swartz RH. Severe hypomagnesemia associated with reversible subacute ataxia and cerebellar hyperintensities on MRI. The neurologist 2012;18(4):223-225. doi: 10.1097/NRL.0b013e31825bbf07

316. Bontadelli J, Müntener D. Posterior reversible encephalopathy syndrome with predominant brainstem involvement: A case report. Cerebrovascular Diseases 2012;33:137-138. doi: 10.1159/000339538

317. Boland T, Strause J, Hu M, Santamaria D, Liang TW, Kremens D, Sergott R, Moussouttas M. Posterior Reversible Encephalopathy Syndrome Presenting as Opsoclonus-Myoclonus. Neuro-ophthalmology (Aeolus Press) 2012;36(4):149-152. doi: 10.3109/01658107.2012.667186

318. Yi JH, Ha SH, Kim YK, Choi EM. Posterior reversible encephalopathy syndrome in an untreated hypertensive patient after spinal surgery under general anesthesia -A case report. Korean journal of anesthesiology 2011;60(5):369-372. doi: 10.4097/kjae.2011.60.5.369

319. Yasuhara T, Tokunaga K, Hishikawa T, Ono S, Miyoshi Y, Sugiu K, Date I. Posterior reversible encephalopathy syndrome. Journal of clinical neuroscience : official journal of the Neurosurgical Society of Australasia 2011;18(3):406-409. doi: 10.1016/j.jocn.2010.06.011

320. Wirojtananugoon C, Laothamatas J. Posterior reversible encephalopathy syndrome with obstructive hydrocephalus. A case report. The neuroradiology journal 2011;24(2):258-263. doi: 10.1177/197140091102400215

321. Suri V, Agarwal R, Jadhao N, Ahuja GK. Cortical blindness after contrast-enhanced CT scan in a patient of sarcoidosis - Is it related to posterior reversible encephalopathy syndrome? Ann Indian Acad Neurol 2011;14(4):298-300. doi: 10.4103/0972-2327.91956

322. Sanchez-Cuadrado I, Lassaletta L, Royo A, Cerdeno V, Roda JM, Gavilan J. Reversible posterior leukoencephalopathy syndrome after lateral skull base surgery. Otology & neurotology : official publication of the American Otological Society, American Neurotology Society [and] European Academy of Otology and Neurotology 2011;32(5):838-840. doi: 10.1097/MAO.0b013e31821f1b95

323. Qin W, Tan CY, Huang X, Huang Z, Tao Y, Fu P. Rapamycin-induced posterior reversible encephalopathy in a kidney transplantation patient. International urology and nephrology 2011;43(3):913-916. doi: 10.1007/s11255-010-9757-0

324. Ogawa E, Sakakibara R, Tateno F, Kishi M, Nakagami T, Terada H. A case of brainstem hypertensive encephalopathy. American Journal of Case Reports 2011;12:95-97. doi: 10.12659/AJCR.881909

325. Negro A, De Berti G, Maggi M, Santi R, Grasselli C, Rossi E. Hypertension-induced posterior reversible encephalopathy syndrome as the presentation of progressive bilateral renal artery stenosis. Journal of cardiology cases 2011;4(3):e168-e171. doi: 10.1016/j.jccase.2011.08.008

326. Morlacchi LC, Rossetti V, DiPasquale M, Salomoni G, Pappalettera M, Tarsia P. Early development of posterior reversible encephalopathy syndrome post lung transplantation. European Respiratory Journal 2011;38.

327. Moosa AN, Eagam M, Moodley M. Reversible brainstem edema due to hypertensive encephalopathy in an 8-year-old girl. Journal of child neurology 2011;26(8):1033-1035. doi: 10.1177/0883073811399802

328. Monteiro C, Sá N, Almeida I, Dias D, Santos E. Posterior reversible encephalopathy syndrome: A flare of systemic lupus erythematosus. European Journal of Neurology 2011;18:586. doi: 10.1111/j.1468-1331.2011.03552.x

329. Lou E, Turner S, Sumrall A, Reardon DA, Desjardins A, Peters KB, Sampson JH, Friedman HS, Vredenburgh JJ. Bevacizumab-induced reversible posterior leukoencephalopathy syndrome and successful retreatment in a patient with glioblastoma. Journal of clinical oncology : official journal of the American Society of Clinical Oncology 2011;29(28):e739-742. doi: 10.1200/jco.2011.36.1865

330. Iwama M, Takahashi H, Takagi R, Hiraoka M. Permanent bilateral cortical blindness due to reversible posterior leukoencephalopathy syndrome. Journal of Nippon Medical School = Nippon Ika Daigaku zasshi 2011;78(3):184-188.

331. Incecik F, Herguner MO, Altunbasak S, Yildizdas D. Reversible posterior encephalopathy syndrome due to intravenous immunoglobulin in a child with Guillain-Barre syndrome. Journal of pediatric neurosciences 2011;6(2):138-140. doi: 10.4103/1817-1745.92841

332. Horie N, Morikawa M, Kitagawa N, Nagata I. Cerebellar variant of posterior reversible encephalopathy syndrome (PRES) after coil embolization for the hemorrhagic dissecting aneurysm. Acta neurochirurgica 2011;153(5):1143-1144. doi: 10.1007/s00701-010-0883-3

333. Gratton D, Szapary P, Goyal K, Fakharzadeh S, Germain V, Saltiel P. Reversible posterior leukoencephalopathy syndrome in a patient treated with ustekinumab: case report and review of the literature. Archives of dermatology 2011;147(10):1197-1202. doi: 10.1001/archdermatol.2011.161

334. Choh NA, Jehangir M, Rasheed M, Mira T, Ahmad I, Choh S. Involvement of the cervical cord and medulla in posterior reversible encephalopathy syndrome. Annals of Saudi medicine 2011;31(1):90-92. doi: 10.4103/0256-4947.75790

335. Aridon P, Ragonese P, Mazzola MA, Quintini G, Lo Re M, Talamanca S, Terruso V, D'Amelio M, Savettieri G. Reversible posterior leukoencephalopathy syndrome in a patient with thrombotic thrombocytopenic purpura. Neurological sciences : official journal of the Italian Neurological Society and of the Italian Society of Clinical Neurophysiology 2011;32(3):469-472. doi: 10.1007/s10072-010-0465-4

336. Aradillas E, Arora R, Gasperino J. Methotrexate-induced posterior reversible encephalopathy syndrome. Journal of clinical pharmacy and therapeutics 2011;36(4):529-536. doi: 10.1111/j.1365-2710.2010.01207.x

337. Agro FE, Doyle DJ, Carassiti M, Antonelli S. A case of posterior reversible encephalopathy syndrome in a 52 year old woman after cardiac arrest. Journal of clinical anesthesia 2011;23(4):310-313. doi: 10.1016/j.jclinane.2010.06.013

338. Zito JA, Lee CC, Johnson S, Singer A, Vacirca J. Reversible posterior leukoencephalopathy syndrome after rituximab. The American journal of emergency medicine 2010;28(4):537.e531-532. doi: 10.1016/j.ajem.2009.04.019

339. Yilmaz S, Gokben S, Arikan C, Calli C, Serdaroglu G. Reversibility of cytotoxic edema in tacrolimus leukoencephalopathy. Pediatr Neurol 2010;43(5):359-362. doi: 10.1016/j.pediatrneurol.2010.05.021

340. Vijayalakshmi B, Sethna F, Manford M, Lees CC. Posterior reversible encephalopathy syndrome in a patient with HELLP syndrome complicating a triploid pregnancy. The journal of maternal-fetal & neonatal medicine : the official journal of the European Association of Perinatal Medicine, the Federation of Asia and Oceania Perinatal Societies, the International Society of Perinatal Obstet 2010;23(8):938-943. doi: 10.3109/14767050903317690

341. Sathyanarayanan V, Razak A, Narayan G, Prabhu M, Ramachandran B, Ranjini K, Vidya M, Joshi K. Posterior reversible encephalopathy syndrome in a patient with hepatitis B induced type 1 membranoproliferative glomerulonephritis. Clinical and experimental nephrology 2010;14(6):614-618. doi: 10.1007/s10157-010-0331-y

342. Sanchez-Carteyron A, Alarcia R, Ara JR, Martin J. Posterior reversible encephalopathy syndrome after rituximab infusion in neuromyelitis optica. Neurology 2010;74(18):1471-1473. doi: 10.1212/WNL.0b013e3181dc1af3

343. Patel AJ, Fox BD, Fulkerson DH, Yallampalli S, Illner A, Whitehead WE, Curry DJ, Luerssen TG, Jea A. Posterior reversible encephalopathy syndrome during posterior fossa tumor resection in a child. Journal of neurosurgery Pediatrics 2010;6(4):377-380. doi: 10.3171/2010.8.Peds10110

344. Park JH, Kim SM, Shin HW, An SJ. Hypertensive brainstem encephalopathy involving deep supratentorial regions: does only blood pressure matter? Neurology international 2010;2(1):e9. doi: 10.4081/ni.2010.e9

345. Oda N, Kato TS, Hanatani A, Niwaya K, Nakatani T, Ishibashi-Ueda H, Kitamura S, Hashimura K, Kitakaze M, Komamura K. Reversible posterior leukoencephalopathy syndrome (RPLS) in a heart transplant recipient treated by substitution of cyclosporine A with tacrolimus. Internal medicine (Tokyo, Japan) 2010;49(11):1013-1016.

346. Nakabou M, Kai T, Maeshima T, Kanamasa K. Hypertensive encephalopathy in patients with chronic renal failure caused by stopping antihypertensive agents: a report of two cases. Clinical and experimental nephrology 2010;14(3):256-262. doi: 10.1007/s10157-009-0252-9

347. Nagato M, Takahashi Y, Yoshioka M, Nambu M. A case of hypertensive encephalopathy with extensive spinal lesions on MRI. Brain & development 2010;32(7):598-601. doi: 10.1016/j.braindev.2009.07.002

348. Malbora B, Avci Z, Donmez F, Alioglu B, Baskin E, Alehan F, Ozbek N. Posterior reversible leukoencephalopathy syndrome in children with hematologic disorders. Turkish Journal of Hematology 2010;27(3):168-176. doi: 10.5152/tjh.2010.24

349. Maeda T, Kikuchi E, Matsumoto K, Yazawa S, Hagiuda J, Miyajima A, Nakagawa K, Fujiwara H, Hoshino H, Oya M. Gemcitabine and cisplatin chemotherapy induced reversible posterior leukoencephalopathy syndrome in a bladder cancer patient. International journal of clinical oncology 2010;15(5):508-511. doi: 10.1007/s10147-010-0068-3

350. Kimura T, Iio K, Imai E, Rakugi H, Isaka Y, Hayashi T. Exercise-induced acute kidney injury with reversible posterior leukoencephalopathy syndrome. Clinical and experimental nephrology 2010;14(2):173-175. doi: 10.1007/s10157-009-0229-8

351. Kheir JN, Lawlor MW, Ahn ES, Lehmann L, Riviello JJ, Silvera VM, McManus M, Folkerth RD. Neuropathology of a fatal case of posterior reversible encephalopathy syndrome. Pediatric and developmental pathology : the official journal of the Society for Pediatric Pathology and the Paediatric Pathology Society 2010;13(5):397-403. doi: 10.2350/09-04-0634-cr.1

352. Kaut O, Kovacs A, Okulla T, Urbach H, Klockgether T. Reversible multifocal leukoencephalopathy associated with a nocturnal blood pressure non-dipper pattern. Blood pressure 2010;19(4):267-269. doi: 10.3109/08037051003718390

353. Katano K, Kakuchi Y, Nakashima A, Nakahama K, Kawano M. Apparent diffusion coefficient map based on diffusion-weighted magnetic resonance imaging is useful in diagnosing the brainstem variant of reversible posterior leukoencephalopathy syndrome with uremia. Clinical and experimental nephrology 2010;14(5):479-482. doi: 10.1007/s10157-010-0293-0

354. Kang SY, Kang JH, Choi JC, Lee JS. Posterior reversible encephalopathy syndrome in a patient with acute intermittent porphyria. Journal of neurology 2010;257(4):663-664. doi: 10.1007/s00415-009-5415-9

355. Heo S, Cho HJ, Jeon IS. A case of posterior reversible encephalopathy syndrome in a child with myelodysplastic syndrome following allogenic bone marrow transplantation. Pediatric hematology and oncology 2010;27(1):59-64. doi: 10.3109/08880010903420661

356. Harnisch E, Leertouwer T, Cransberg K, Kist-van Holthe JE. A 3-year old girl with seizures, hypokalemia and metabolic alkalosis. BMJ case reports 2010;2010. doi: 10.1136/bcr.11.2009.2500

357. Forteza A, Echeverria Y, Haussen DC, Gutierrez J, Wiley E, De Gusmao C. Cerebral vasomotor reactivity monitoring in posterior reversible encephalopathy syndrome. BMJ case reports 2010;2010. doi: 10.1136/bcr.10.2009.2345

358. Baytan B, Ozdemir O, Demirkaya M, Evim MS, Gunes AM. Reversible Posterior Leukoencephalopathy Induced by Cancer Chemotherapy. Pediatric Neurology 2010;43(3):197-201. doi: 10.1016/j.pediatrneurol.2010.05.005

359. Zamvar V, Sugarman ID, Tawfik RF, Macmullen-Price J, Puntis JW. Posterior reversible encephalopathy syndrome following infliximab infusion. Journal of pediatric gastroenterology and nutrition 2009;48(1):102-105. doi: 10.1097/MPG.0b013e31818aedb4

360. Yerdelen D, Giray S, Tan M, Yildirim T. Hypertensive encephalopathy with atypical MRI leukoencephalopathy affecting brain stem and cerebellum. Acta neurologica Belgica 2009;109(2):142-145.

361. Reutenauer S, Albucher JF, Pariente J, Dumas H, Milioto O, Attal M, Recher C, Huguet F. Posterior reversible encephalopathy syndrome: two cases in young adults with acute lymphoblastic leukemia. Leukemia research 2009;33(6):e1-3. doi: 10.1016/j.leukres.2008.09.003

362. Oytun Bayrak A, Karbek B, Cengiz K, Incesu L, Öztaş G, Cengiz N. A case report of reversible posterior leukoencephalopathy syndrome associated with severe neurologic dysfunction. Journal of Neurological Sciences 2009;26(1):83-86.

363. Ogaki K, Fukae J, Noda K, Fujishima K, Hattori N, Okuma Y. Blurred Vision With Acute Hypertension Indicating Hypertensive Brainstem Encephalopathy-Case Report. Neurologia Medico-Chirurgica 2009;49(12):625-630. doi: 10.2176/nmc.49.625

364. Ocvirk J, Boc M, Rebersek M, Ros T. Cisplatin-induced non-convulsive posterior reversible encephalopathy syndrome in a 41-year-old woman with metastatic malignant melanoma. Radiology and Oncology 2009;43(2):120-125. doi: 10.2478/v10019-009-0005-0

365. Ngow HA, Wan Khairina WM, Hamidon BB. An unsual finding of brain magnetic resonance imaging in a hypertensive patient. Neurology international 2009;1(1):e3. doi: 10.4081/ni.2009.e3

366. Medici E, Arabi S, Sebastianelli M, Pirani O, Giaccaglini E. A case of hypertensive encephalopathy with prominent brainstem presentation. Neurological sciences : official journal of the Italian Neurological Society and of the Italian Society of Clinical Neurophysiology 2009;30(2):133-135. doi: 10.1007/s10072-009-0018-x

367. Lee SY, Kim SH, Lee SH, Baek HJ, Shon HS, Kim SS. Serial MR spectroscopy in relapsing reversible posterior leukoencephalopathy syndrome. The neurologist 2009;15(6):338-341. doi: 10.1097/NRL.0b013e3181914af6

368. Lapuyade B, Sibon I, Jeanin S, Dousset V. Neurological picture. Spinal cord involvement in posterior reversible encephalopathy syndrome. Journal of neurology, neurosurgery, and psychiatry 2009;80(1):35. doi: 10.1136/jnnp.2008.154781

369. Lapuyade B, Sibon I, Jeanin S, Dousset V. Spinal cord involvement in posterior reversible encephalopathy syndrome. Journal of Neurology Neurosurgery and Psychiatry 2009;80(1):35-35. doi: 10.1136/jnnp.2008.154781

370. Karakis I, Macdonald JA, Stefanidou M, Kase CS. Clinical and radiological features of brainstem variant of hypertensive encephalopathy. Journal of vascular and interventional neurology 2009;2(2):172-176.

371. Hodnett P, Coyle J, O'Regan K, Maher MM, Fanning N. PRES (posterior reversible encephalopathy syndrome), a rare complication of tacrolimus therapy. Emergency radiology 2009;16(6):493-496. doi: 10.1007/s10140-008-0782-6

372. Dzudie A, Boissonnat P, Roussoulieres A, Cakmak, Mosbah K, Bejui FT, Obadia JF, Sebbag L. Cyclosporine-related posterior reversible encephalopathy syndrome after heart transplantation: should we withdraw or reduce cyclosporine?: case reports. Transplant Proc 2009;41(2):716-720. doi: 10.1016/j.transproceed.2009.01.041

373. Decker DA, Falchook AD, Yachnis AT, Waters MF. Radiographic and pathologic findings in an atypical brainstem variant of reversible posterior leukoencephalopathy syndrome. The neurologist 2009;15(6):364-366. doi: 10.1097/NRL.0b013e3181951ac7

374. Chen TY, Lee HJ, Wu TC, Tsui YK. MR imaging findings of medulla oblongata involvement in posterior reversible encephalopathy syndrome secondary to hypertension. AJNR American journal of neuroradiology 2009;30(4):755-757. doi: 10.3174/ajnr.A1337

375. Chawla R, Smith D, Marik PE. Near fatal posterior reversible encephalopathy syndrome complicating chronic liver failure and treated by induced hypothermia and dialysis: a case report. J Med Case Rep 2009;3:6623. doi: 10.1186/1752-1947-3-6623

376. Briganti C, Caulo M, Notturno F, Tartaro A, Uncini A. Asymptomatic spinal cord involvement in posterior reversible encephalopathy syndrome. Neurology 2009;73(18):1507-1508. doi: 10.1212/WNL.0b013e3181bf98c9

377. Benziada-Boudour A, Schmitt E, Kremer S, Foscolo S, Riviere AS, Tisserand M, Boudour A, Bracard S. Posterior reversible encephalopathy syndrome: a case of unusual diffusion-weighted MR images. Journal of neuroradiology Journal de neuroradiologie 2009;36(2):102-105. doi: 10.1016/j.neurad.2008.08.003

378. Sanjay KM, Partha PC. The posterior reversible encephalopathy syndrome. Indian journal of pediatrics 2008;75(9):953-955. doi: 10.1007/s12098-008-0168-5

379. Ridolfo AL, Resta F, Milazzo L, Caramma I, Matacena G, Antinori S, Galli M. Reversible posterior leukoencephalopathy syndrome in 2 HIV-infected patients receiving antiretroviral therapy. Clinical infectious diseases : an official publication of the Infectious Diseases Society of America 2008;46(2):e19-22. doi: 10.1086/524740

380. Pratap JN, Down JF. Posterior reversible encephalopathy syndrome: a report of a case with atypical features. Anaesthesia 2008;63(11):1245-1248. doi: 10.1111/j.1365-2044.2008.05587.x

381. Park J, Choi SH, Kwon JH, Chung HC, Lee JS. Atypical reversible posterior leukoencephalopathy syndrome in a chronic haemodialysis patient with severe hypertension. Nephrology (Carlton, Vic) 2008;13(6):542-543. doi: 10.1111/j.1440-1797.2008.00951.x

382. Morelli N, Gori S, Michelassi MC, Falorni M, Cafforio G, Bianchi MC, Cosottini M, Orlandi G, Murri L, Tartaglione A. Atypical posterior reversible encephalopathy syndrome in puerperium. European neurology 2008;59(3-4):195-197. doi: 10.1159/000114044

383. Milia A, Moller J, Pilia G, Mascia MG, Marchi P, Mura M, Marrosu MG. Spinal cord involvement during hypertensive encephalopathy: clinical and radiological findings. Journal of neurology 2008;255(1):142-143. doi: 10.1007/s00415-008-0698-9

384. McCarron MO, McKinstry CS. Vanishing brainstem edema. Journal of stroke and cerebrovascular diseases : the official journal of National Stroke Association 2008;17(3):156-157. doi: 10.1016/j.jstrokecerebrovasdis.2007.12.006

385. Lim MH, Kim DW, Cho HS, Lee HJ, Kim HJ, Park KJ, Chang SH, Park DJ. Isolated cerebellar reversible leukoencephalopathy syndrome in a patient with end stage renal disease. Internal medicine (Tokyo, Japan) 2008;47(1):43-45.

386. Legriel S, Bruneel F, Spreux-Varoquaux O, Birenbaum A, Chadenat ML, Mignon F, Abbosh N, Henry-Lagarrigue M, D'Allonnes LR, Guezennec P, Troche G, Bedos JP. Lysergic acid amide-induced posterior reversible encephalopathy syndrome with status epilepticus. Neurocritical Care 2008;9(2):247-252. doi: 10.1007/s12028-008-9096-5

387. Lee SY, Dinesh SK, Thomas J. Hypertension-induced reversible posterior leukoencephalopathy syndrome causing obstructive hydrocephalus. Journal of clinical neuroscience : official journal of the Neurosurgical Society of Australasia 2008;15(4):457-459. doi: 10.1016/j.jocn.2006.12.019

388. Kastrup O, Diener HC. TNF-antagonist etanercept induced reversible posterior leukoencephalopathy syndrome. Journal of neurology 2008;255(3):452-453. doi: 10.1007/s00415-008-0732-y

389. Hourani R, Abboud M, Hourani M, Khalifeh H, Muwakkit S. L-asparaginase-induced posterior reversible encephalopathy syndrome during acute lymphoblastic leukemia treatment in children. Neuropediatrics 2008;39(1):46-50. doi: 10.1055/s-2008-1076740

390. Hamilton BE, Nesbit GM. Delayed CSF enhancement in posterior reversible encephalopathy syndrome. AJNR American journal of neuroradiology 2008;29(3):456-457. doi: 10.3174/ajnr.A0926

391. El Maalouf G, Mitry E, Lacout A, Lievre A, Rougier P. Isolated brainstem involvement in posterior reversible leukoencephalopathy induced by bevacizumab. Journal of neurology 2008;255(2):295-296. doi: 10.1007/s00415-008-0692-2

392. El Karoui K, Le Quintrec M, Dekeyser E, Servais A, Hummel A, Fadel F, Fakhouri F. Posterior reversible encephalopathy syndrome in systemic lupus erythematosus. Nephrology, dialysis, transplantation : official publication of the European Dialysis and Transplant Association - European Renal Association 2008;23(2):757-763. doi: 10.1093/ndt/gfm811

393. Bhagavati S, Chum F, Choi J. Hypertensive encephalopathy presenting with isolated brain stem and cerebellar edema. Journal of neuroimaging : official journal of the American Society of Neuroimaging 2008;18(4):454-456. doi: 10.1111/j.1552-6569.2007.00213.x

394. Bhagavati S, Choi J. Atypical cases of posterior reversible encephalopathy syndrome. Clinical and MRI features. Cerebrovascular diseases (Basel, Switzerland) 2008;26(5):564-566. doi: 10.1159/000164556

395. Bas DF, Oguz KK, Topcuoglu MA. Atypical reversible posterior leukoencephalopathy syndrome in thrombotic thrombocytopenic purpura. Internal medicine (Tokyo, Japan) 2008;47(21):1931-1934.

396. Uchino M, Haga D, Nomoto J, Mito T, Kuramitsu T. Brainstem involvement in hypertensive encephalopathy: a report of two cases and literature review. European neurology 2007;57(4):223-226. doi: 10.1159/000100015

397. Tanioka R, Yamamoto Y, Sakai M, Makie T, Mori M, Uehira T, Shirasaka T. Convalescence of atypical reversible posterior leukoencephalopathy syndrome in human immunodeficiency virus infection. The journal of medical investigation : JMI 2007;54(1-2):191-194.

398. Skelton MR, Goldberg RM, O'Neil BH. A case of oxaliplatin-related posterior reversible encephalopathy syndrome. Clinical colorectal cancer 2007;6(5):386-388. doi: 10.3816/CCC.2007.n.009

399. Seet RCS, Lim ECH. Hypertensive brainstem encephalopathy. Circulation 2007;115(9):E310-E311. doi: 10.1161/circulationaha.106.653618

400. Sasayama D, Shimojima Y, Gono T, Kaneko K, Matsuda M, Ikeda S. Henoch-Schonlein purpura nephritis complicated by reversible posterior leukoencephalopathy syndrome. Clinical rheumatology 2007;26(10):1761-1763. doi: 10.1007/s10067-006-0502-0

401. Saito B, Nakamaki T, Nakashima H, Usui T, Hattori N, Kawakami K, Tomoyasu S. Reversible posterior leukoencephalopathy syndrome after repeat intermediate-dose cytarabine chemotherapy in a patient with acute myeloid leukemia. American journal of hematology 2007;82(4):304-306. doi: 10.1002/ajh.20772

402. Saeed MU, Dacuycuy MA, Kennedy DJ. Posterior reversible encephalopathy syndrome in HIV patients: case report and review of the literature. AIDS (London, England) 2007;21(6):781-782. doi: 10.1097/QAD.0b013e3280b07795

403. Punaro M, Abou-Jaoude P, Cimaz R, Ranchin B. Unusual neurologic manifestations (II): posterior reversible encephalopathy syndrome (PRES) in the context of juvenile systemic lupus erythematosus. Lupus 2007;16(8):576-579. doi: 10.1177/0961203307079811

404. O'Riordan S, McGuigan C, Stevens J, Chapman N, Ball J. Reversible hypertensive cerebellar encephalopathy and hydrocephalus. Journal of neurology, neurosurgery, and psychiatry 2007;78(9):1008-1009. doi: 10.1136/jnnp.2006.107672

405. Norman JK, Parke JT, Wilson DA, McNall-Knapp RY. Reversible posterior leukoencephalopathy syndrome in children undergoing induction therapy for acute lymphoblastic leukemia. Pediatric blood & cancer 2007;49(2):198-203. doi: 10.1002/pbc.20597

406. Moskowitz A, Nolan C, Lis E, Castro-Malaspina H, Perales MA. Posterior reversible encephalopathy syndrome due to sirolimus. Bone Marrow Transplant 2007;39(10):653-654. doi: 10.1038/sj.bmt.1705659

407. Kitabayashi Y, Hamamoto Y, Hirosawa R, Narumoto J, Fukui K. Postpartum catatonia associated with atypical posterior reversible encephalopathy syndrome. The Journal of neuropsychiatry and clinical neurosciences 2007;19(1):91-92. doi: 10.1176/jnp.2007.19.1.91a

408. Kim JS, Lee KS, Lim SC, Ahn JY, Song IU, Kim YI, Kim BS, Kim HT. Reversible posterior leukoencephalopathy syndrome in a patient with multiple system atrophy: a possible association with oral midodrine treatment. Movement disorders : official journal of the Movement Disorder Society 2007;22(7):1043-1046. doi: 10.1002/mds.21461

409. Keyserling HF, Provenzale JM. Atypical imaging findings in a near-fatal case of posterior reversible encephalopathy syndrome in a child. AJR American journal of roentgenology 2007;188(1):219-221. doi: 10.2214/ajr.05.0328

410. Kang SY, Choi JC, Kang JH. Two cases of hypertensive encephalopathy involving the brainstem. Journal of clinical neurology (Seoul, Korea) 2007;3(1):50-52. doi: 10.3988/jcn.2007.3.1.50

411. Irvin W, MacDonald G, Smith JK, Kim WY. Dexamethasone-induced posterior reversible encephalopathy syndrome. Journal of clinical oncology : official journal of the American Society of Clinical Oncology 2007;25(17):2484-2486. doi: 10.1200/jco.2007.10.9991

412. Hagan IG, Burney K. Radiology of recreational drug abuse. Radiographics : a review publication of the Radiological Society of North America, Inc 2007;27(4):919-940. doi: 10.1148/rg.274065103

413. Doelken M, Lanz S, Rennert J, Alibek S, Richter G, Doerfler A. Differentiation of cytotoxic and vasogenic edema in a patient with reversible posterior leukoencephalopathy syndrome using diffusion-weighted MRI. Diagnostic and interventional radiology (Ankara, Turkey) 2007;13(3):125-128.

414. Chowdhary S, Chamberlain MC. Neuroradiographic variant of posterior reversible leukoencephalopathy. Journal of Neuro-Oncology 2007;82(1):111-112. doi: 10.1007/s11060-006-9243-9

415. Bink A, Weidauer S, Hermans M, Kell C, Lanfermann H. Reversible bilateral pyramidal tract lesions after hypertensive crisis and cerebral seizures. Journal of neuroradiology Journal de neuroradiologie 2007;34(5):340-343. doi: 10.1016/j.neurad.2007.09.002

416. Yokobori S, Yokota H, Yamamoto Y. Pediatric posterior reversible leukoencephalopathy syndrome and NSAID-induced acute tubular interstitial nephritis. Pediatr Neurol 2006;34(3):245-247. doi: 10.1016/j.pediatrneurol.2005.07.012

417. Soysal DD, Caliskan M, Aydin K, Nayir A, Karabocuoglu M, Citak A, Uzel N. Isolated cerebellar involvement in a case of posterior reversible leukoencephalopathy. Clinical radiology 2006;61(11):983-986. doi: 10.1016/j.crad.2006.07.001

418. Narbone MC, Musolino R, Granata F, Mazzu I, Abbate M, Ferlazzo E. PRES: posterior or potentially reversible encephalopathy syndrome? Neurological sciences : official journal of the Italian Neurological Society and of the Italian Society of Clinical Neurophysiology 2006;27(3):187-189. doi: 10.1007/s10072-006-0667-y

419. Min LL, Zwerling J, Ocava LC, Chen IHA, Putterman C. Reversible posterior leukoencephalopathy in connective tissue diseases. Seminars in Arthritis and Rheumatism 2006;35(6):388-395. doi: 10.1016/j.semarthrit.2006.01.003

420. Machinis TG, Fountas KN, Dimopoulos VG, Troup EC. Spontaneous posterior fossa hemorrhage associated with low-molecular weight heparin in an adolescent recently diagnosed with posterior reversible encephalopathy syndrome: case report and review of the literature. Child's nervous system : ChNS : official journal of the International Society for Pediatric Neurosurgery 2006;22(11):1487-1491. doi: 10.1007/s00381-006-0155-y

421. Lin KL, Hsu WC, Wang HS, Lui TN. Hypertension-induced cerebellar encephalopathy and hydrocephalus in a male. Pediatr Neurol 2006;34(1):72-75. doi: 10.1016/j.pediatrneurol.2005.07.005

422. Kur JK, Esdaile JM. Posterior reversible encephalopathy syndrome - An underrecognized manifestation of systemic lupus erythematosus. Journal of Rheumatology 2006;33(11):2178-2183.

423. Kaleyias J, Faerber E, Kothare SV. Tacrolimus induced subacute cerebellar ataxia. European journal of paediatric neurology : EJPN : official journal of the European Paediatric Neurology Society 2006;10(2):86-89. doi: 10.1016/j.ejpn.2006.01.002

424. Gamanagatti S, Subramanian S. Hypertensive encephalopathy: isolated pons involvement mimicking central pontine myelinolysis. Korean journal of radiology 2006;7(3):218-219. doi: 10.3348/kjr.2006.7.3.218

425. Ferraz-Filho JR, Rocha-Filho JA, Bichuette TF, de Albuquerque RCAP, Sanchez RA, Souza AS. Studying the evolution and the magnetic resonance findings of reversible posterior leukoencephalopathy in children. Arquivos De Neuro-Psiquiatria 2006;64(3B):718-722. doi: 10.1590/s0004-282x2006000500003

426. Doi Y, Kimura F, Fujiyama T, Fujimura C, Nishina T, Sato T, Hosokawa T, Uehara H, Ishida S, Hanafusa T. Hypertensive Brainstem encephalopathy without parieto-occipital lesion - Two case reports. Neurologia Medico-Chirurgica 2006;46(2):75-79. doi: 10.2176/nmc.46.75

427. Albini TA, Lakhanpal RR, Foroozan R, Lopez GA, McPherson AR. Retinopathy and choroidopathy as the initial signs of hypertensive brainstem encephalopathy. Archives of ophthalmology (Chicago, Ill : 1960) 2006;124(12):1784-1786. doi: 10.1001/archopht.124.12.1784

428. Wakely SL, Ditchfield A. Hypertensive encephalopathy: a rare case of isolated pons involvement. Clinical Radiology Extra 2005;60(5):E53-E56. doi: 10.1016/j.cradex.2005.01.001

429. Shrivastava A, Yousaf N, Vaidhyanath R. Posterior reversible encephalopathy syndrome. Hospital medicine (London, England : 1998) 2005;66(1):56-57. doi: 10.12968/hmed.2005.66.1.17541

430. Seneviratne J, Brotchie P, Gates P, Talman P. An unusual case of hypertensive encephalopathy. Journal of clinical neuroscience : official journal of the Neurosurgical Society of Australasia 2005;12(3):323-326. doi: 10.1016/j.jocn.2004.06.003

431. Rangi PS, Partridge WJ, Newlands ES, Waldman AD. Posterior reversible encephalopathy syndrome: a possible late interaction between cytotoxic agents and general anaesthesia. Neuroradiology 2005;47(8):586-590. doi: 10.1007/s00234-005-1376-6

432. Pasupuleti DV, Miranda M, Vattipally V. Case report: posterior reversible encephalopathy syndrome. American family physician 2005;72(12):2430, 2434, 2496.

433. Parisaei M, Derwig I, Yoon J, Erskine KJ, Jarman PR. Posterior reversible leukoencephalopathy or in a case of postpartum eclampsia. American Journal of Obstetrics and Gynecology 2005;193(3):885-886. doi: 10.1016/j.ajog.2005.02.077

434. Ono Y, Manabe Y, Hamakawa Y, Murakami T, Omori N, Hayashi Y, Abe K. Localized lesions on MRI in a case of hypertensive brainstem encephalopathy. Internal medicine (Tokyo, Japan) 2005;44(9):1002-1005. doi: 10.2169/internalmedicine.44.1002

435. Ohtomo Y, Takada M, Fujinaga S, Murakami H, Yamashiro Y. Hypertensive encephalopathy in a boy with biopsy-proven acute post-streptococcal glomerulonephritis. Pediatrics international : official journal of the Japan Pediatric Society 2005;47(3):323-325. doi: 10.1111/j.1442-200x.2005.02071.x

436. Negro A, Zuccoli G, Regolisti G, Mastrangeli S, Rossi E. Reversible posterior leukoencephalopathy associated with postpartum HELLP syndrome. European Journal of Internal Medicine 2005;16(4):291-293. doi: 10.1016/j.ejim.2004.11.010

437. Mehall JR, Leach JL, Merrill WH. Posterior reversible encephalopathy syndrome after nontransplant cardiac surgery. The Journal of thoracic and cardiovascular surgery 2005;130(5):1473-1474. doi: 10.1016/j.jtcvs.2005.07.030

438. Kitaguchi H, Tomimoto H, Miki Y, Yamamoto A, Terada K, Satoi H, Kanda M, Fukuyama H. A brainstem variant of reversible posterior leukoencephalopathy syndrome. Neuroradiology 2005;47(9):652-656. doi: 10.1007/s00234-005-1399-z

439. Kanazawa M, Sanpei K, Kasuga K. Recurrent hypertensive brainstem encephalopathy. Journal of Neurology Neurosurgery and Psychiatry 2005;76(6):888-890. doi: 10.1136/jnnp.2004.059543

440. Ito S, Ito T, Tamura N, Uchiyama T, Mori M, Hattori T. Chronic hypertensive encephalopathy showing only headache: report of a case with long-standing brain MR abnormalities suggesting extensive vasogenic edema. European neurology 2005;53(4):220-222. doi: 10.1159/000086736

441. Adamson DC, Dimitrov DF, Bronec PR. Upward transtentorial herniation, hydrocephalus, and cerebellar edema in hypertensive encephalopathy. The neurologist 2005;11(3):171-175. doi: 10.1097/01.nrl.0000159982.63592.9f

442. Ozcakar ZB, Ekim M, Fitoz S, Teber S, Hizel S, Acar B, Yuksel S, Yalcinkaya F. Hypertension induced reversible posterior leukoencephalopathy syndrome: a report of two cases. European journal of pediatrics 2004;163(12):728-730. doi: 10.1007/s00431-004-1532-8

443. Nagata M, Maeda M, Tsukahara H, Maier SE, Takeda K. Brain stem hypertensive encephalopathy evaluated by line scan diffusion-weighted imaging. AJNR American journal of neuroradiology 2004;25(5):803-806.

444. Karasawa S, Kawanami T, Kimura H, Kurita K, Kato T. An unusual case of hypertensive encephalopathy involving the brain stem. Internal medicine (Tokyo, Japan) 2004;43(5):448-449.

445. Karampekios SK, Contopoulou E, Basta M, Tzagournissakis M, Gourtsoyiannis N. Hypertensive encephalopathy with predominant brain stem involvement: MRI findings. Journal of human hypertension 2004;18(2):133-134. doi: 10.1038/sj.jhh.1001654

446. Cruz-Flores S, Gondim FDA, Leira EC. Brainstem involvement in hypertensive encephalopathy - Clinical and radiological findings. Neurology 2004;62(8):1417-1419. doi: 10.1212/01.Wnl.0000120668.73677.5f

447. Cruz-Flores S, de Assis Aquino Gondim F, Leira EC. Brainstem involvement in hypertensive encephalopathy: clinical and radiological findings. Neurology 2004;62(8):1417-1419.

448. Chang GY. Hypertensive brainstem encephalopathy. Neurosurgery Quarterly 2004;14(3):179-180. doi: 10.1097/01.wnq.0000135807.77238.75

449. Thambisetty M, Biousse V, Newman NJ. Hypertensive brainstem encephalopathy: clinical and radiographic features. J Neurol Sci 2003;208(1-2):93-99.

450. Nakagawa K, Yamaguchi T, Seida M, Tanaka Y, Yoshino M. Plasma concentrations of atrial and brain natriuretic peptides in a case with hypertensive encephalopathy. Neurological research 2002;24(6):627-630. doi: 10.1179/016164102101200474

451. Kumai Y, Toyoda K, Fujii K, Ibayashi S. Hypertensive encephalopathy extending into the whole brainstem and deep structures. Hypertension research : official journal of the Japanese Society of Hypertension 2002;25(5):797-800.

452. Keswani SC, Wityk R. Don't throw in the towel! A case of reversible coma. Journal of neurology, neurosurgery, and psychiatry 2002;73(1):83-84.

453. Giner V, Fernandez C, Esteban MJ, Galindo MJ, Forner MJ, Guix J, Redon J. Reversible posterior leukoencephalopathy secondary to indinavir-induced hypertensive crisis: a case report. American journal of hypertension 2002;15(5):465-467.

454. Yoshida K, Yamamoto T, Mori K, Maeda M. Reversible posterior leukoencephalopathy syndrome in a patient with hypertensive encephalopathy--case report. Neurol Med Chir (Tokyo) 2001;41(7):364-369.

455. Morello F, Marino A, Cigolini M, Cappellari F. Hypertensive brain stem encephalopathy: clinically silent massive edema of the pons. Neurological sciences : official journal of the Italian Neurological Society and of the Italian Society of Clinical Neurophysiology 2001;22(4):317-320.

456. Drees C, Alkotob L, Hall PM, Krieger D. Reversible pontine edema in hypertension. Neurology 2001;56(5):659. doi: 10.1212/wnl.56.5.659

457. Chu K, Kang DW, Lee SH, Kim M. Diffusion-weighted MR findings in brain stem hypertensive encephalopathy: a possibility of cytotoxic edema? European neurology 2001;46(4):220-222. doi: 10.1159/000050810

458. Oliverio PJ, Restrepo L, Mitchell SA, Tornatore CS, Frankel SR. Reversible tacrolimus-induced neurotoxicity isolated to the brain stem. AJNR American journal of neuroradiology 2000;21(7):1251-1254.

459. de Seze J, Mastain B, Stojkovic T, Ferriby D, Pruvo JP, Destee A, Vermersch P. Unusual MR findings of the brain stem in arterial hypertension. AJNR American journal of neuroradiology 2000;21(2):391-394.

460. Casey SO, Truwit CL. Pontine reversible edema: a newly recognized imaging variant of hypertensive encephalopathy? AJNR American journal of neuroradiology 2000;21(2):243-245.

461. Wang MC, Escott EJ, Breeze RE. Posterior fossa swelling and hydrocephalus resulting from hypertensive encephalopathy: case report and review of the literature. Neurosurgery 1999;44(6):1325-1327.

462. Chang GY, Keane JR. Hypertensive brainstem encephalopathy: three cases presenting with severe brainstem edema. Neurology 1999;53(3):652-654. doi: 10.1212/wnl.53.3.652

463. Bakshi R, Bates VE, Mechtler LL, Kinkel PR, Kinkel WR. Occipital lobe seizures as the major clinical manifestation of reversible posterior leukoencephalopathy syndrome: magnetic resonance imaging findings. Epilepsia 1998;39(3):295-299.

464. Nakano H, Tomita Y, Bandoh K, Miyaoka M. Malignant hypertension associated with obstructive hydrocephalus--case report. Neurol Med Chir (Tokyo) 1997;37(3):265-269.

465. Jones BV, Egelhoff JC, Patterson RJ. Hypertensive encephalopathy in children. AJNR American journal of neuroradiology 1997;18(1):101-106.

466. Jarosz JM, Howlett DC, Cox TC, Bingham JB. Cyclosporine-related reversible posterior leukoencephalopathy: MRI. Neuroradiology 1997;39(10):711-715. doi: 10.1007/s002340050492

467. Katsumata Y, Maehara T, Noda M, Shirouzu I, Shimokawa M, Hiyamuta E. Hypertensive encephalopathy: reversible CT and MR appearance. Radiation medicine 1993;11(4):160-163.
